# Ipriflavone as a non‐steroidal glucocorticoid receptor antagonist ameliorates diabetic cognitive impairment in mice

**DOI:** 10.1111/acel.13572

**Published:** 2022-02-16

**Authors:** Ruifang Nie, Jian Lu, Rui Xu, Juanzhen Yang, Xingyi Shen, Xingnan Ouyang, Danyang Zhu, Yujie Huang, Tong Zhao, Xuejian Zhao, Yin Lu, Minyi Qian, Jiaying Wang, Xu Shen

**Affiliations:** ^1^ Jiangsu Key Laboratory for Pharmacology and Safety Evaluation of Chinese Materia Medica and School of Medicine & Holistic Integrative Medicine Nanjing University of Chinese Medicine Nanjing China

**Keywords:** antagonist, diabetes, diabetic cognitive impairment, glucocorticoid receptor, glucocorticoids, ipriflavone

## Abstract

Diabetic cognitive impairment (DCI) is a common diabetic complication with hallmarks of loss of learning ability and disorders of memory and behavior. Glucocorticoid receptor (GR) dysfunction is a main reason for neuronal impairment in brain of diabetic patients. Here, we determined that ipriflavone (IP) a clinical anti‐osteoporosis drug functioned as a non‐steroidal GR antagonist and efficiently ameliorated learning and memory dysfunction in both type 1 and 2 diabetic mice. The underlying mechanism has been intensively investigated by assay against the diabetic mice with GR‐specific knockdown in the brain by injection of adeno‐associated virus (AAV)‐ePHP‐*si*‐*GR*. IP suppressed tau hyperphosphorylation through GR/PI3K/AKT/GSK3β pathway, alleviated neuronal inflammation through GR/NF‐κB/NLRP3/ASC/Caspase‐1 pathway, and protected against synaptic impairment through GR/CREB/BDNF pathway. To our knowledge, our work might be the first to expound the detailed mechanism underlying the amelioration of non‐steroidal GR antagonist on DCI‐like pathology in mice and report the potential of IP in treatment of DCI.

## Graphical abstract

Schematic diagram illustrating the mechanism underlying the amelioration of IP as a non‐steroidal GR antagonist on diabetic cognitive impairment in mice. IP attenuated tau hyperphosphorylation through GR/PI3K/AKT/GSK3β pathway, ameliorated neuroinflammation through GR/NF‐κB/NLRP3/ASC/Caspase‐1 pathway, and protected against synaptic impairment involving GR/BDNF/TrkB/CREB pathway.

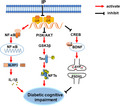

**Abbreviations**
ASC: the adaptor molecule apoptosis‐associated speck‐like protein containing a CARD
CNS: central nervous system
DCI: diabetic cognitive impairment
ER: estrogen receptor
GR: glucocorticoid receptor
IL‐1β: interleukin 1β
IP: ipriflavone
LPS: lipopolysaccharide
LTP: long‐term potentiation
MR: mineralocorticoid receptor
MWM: Morris water maze
NFTs: neurofibrillary tangles
NLRP3: NLR Family Pyrin Domain Containing 3
NOR: novel object recognition
OFT: open filed test
PA: palmitic acid
STZ: streptozotocin
SYN: synaptophysin
T1DM: type‐1 diabetes mellitus
T2DM: type‐2 diabetes mellitus
TNF‐α: tumor necrosis factor α

## 1 INTRODUCTION

Diabetic cognitive impairment (DCI) is a common diabetic complication with hallmarks of loss of learning ability and disorders of memory and behavior (Biessels & Whitmer, 2020), severely deteriorating the life quality of the patients. Diabetes mellitus (DM) is a global epidemic disease affecting more than 422 million people worldwide and 30% of them are suffering from DCI with a higher risk for Alzheimer's disease (AD) than common population (Riederer et al., 2017), while the current available therapy remains extremely limited.

The pathogenesis of DCI is complicated. Hyperglycemia and total insulin deficiency are reported to be mainly responsible for the increased incidence of DCI in patients with type‐1 diabetes mellitus (T1DM), while hyperglycemia and insulin resistance are acknowledged as the key risk factors for DCI in patients with type‐2 diabetes mellitus (T2DM) (Batista et al., 2018). Some typical pathological factors in brain of diabetic mice have been reported. For example, paired‐helical filament tau (PHF‐tau) as the main component of neurofibrillary tangles (NFTs) is observed in the hippocampus of T1DM and T2DM mice (Kim et al., 2009); microglia activation and pro‐inflammatory cytokines elevation are found in the hippocampus of diabetic patients (Gaspar et al., 2016); activation of NF‐κB signaling that is for NLRP3 inflammasome assembly leading to inflammatory cascade and cognitive impairment is identified in T2DM mice (Biessels et al., 2006). Notably, activated microglia have been observed to aggregate surrounding neurons with hyperphosphorylated tau in tauopathy brains, and NF‐κB signaling is activated in isolated microglia from tau transgenic mice, implying the tight relationship between inflammation and tau pathology (Batista et al., 2018; Biessels et al., 2006). Moreover, it has been found that the levels of several neurotransmitters and the protein expression of post‐synaptic membrane are declined in diabetic mice (Hamed, 2017), while inflammation and tau hyperphosphorylation may exacerbate synapse impairment eventually triggering the dysfunction of learning and memory (Liu et al., 2020).

The glucocorticoid receptor (GR) is an evolutionally conserved nuclear receptor superfamily protein (Weikum et al., 2017). In the absence of hormone, GR resides in the cytosol complexed with a variety of proteins (Pedrazzoli et al., 2019). Once activated by hormone, GR will translocate into nucleus to induce the downstream transcription of the target genes initiating varied biological effects (Pedrazzoli et al., 2019). GR has been currently received much attention for its potent role in neurological disorders (Williams & Ghosh, 2020), since it was determined that GR dysfunction rather than hyperglycemia is the main reason for synaptic injury in diabetic patients (Russo et al., 2016). Actually, accumulating evidence has indicated that GR dysfunction evokes inflammation in central nervous system (CNS) thereby damaging hippocampal neurons and reducing the number of dendritic spines (Yi et al., 2017). In addition, elevated levels of GR, GSK3β, and hyperphosphorylated‐tau were also identified in brains of T2DM mice (Dey et al., 2017). All findings have highlighted the association of GR regulation with DCI. Moreover, it was noted that mifepristone (Mife) as a steroidal GR antagonist displayed favorable effect in treating DCI in animal models, although the side effects have limited its clinical utilization (Pedrazzoli et al., 2019). Thus, exploring novel non‐steroidal GR antagonist that targets GR in CNS should be a promising strategy for treating DCI.

Recently, several non‐steroidal GR antagonists have been reported in the amelioration of synaptic deficit and cognitive impairment in mice (Canet et al., 2018). For example, non‐steroidal GR antagonists CORT108297 and CORT113176 (Canet et al., 2018) improved hippocampus synaptic deficits by upregulating the expressions of synaptic marker proteins (PSD95, synaptophysin), indicative of the beneficial effect of GR antagonism on cognitive dysfunction, although the detailed mechanisms are much needed.

**FIGURE 1:**
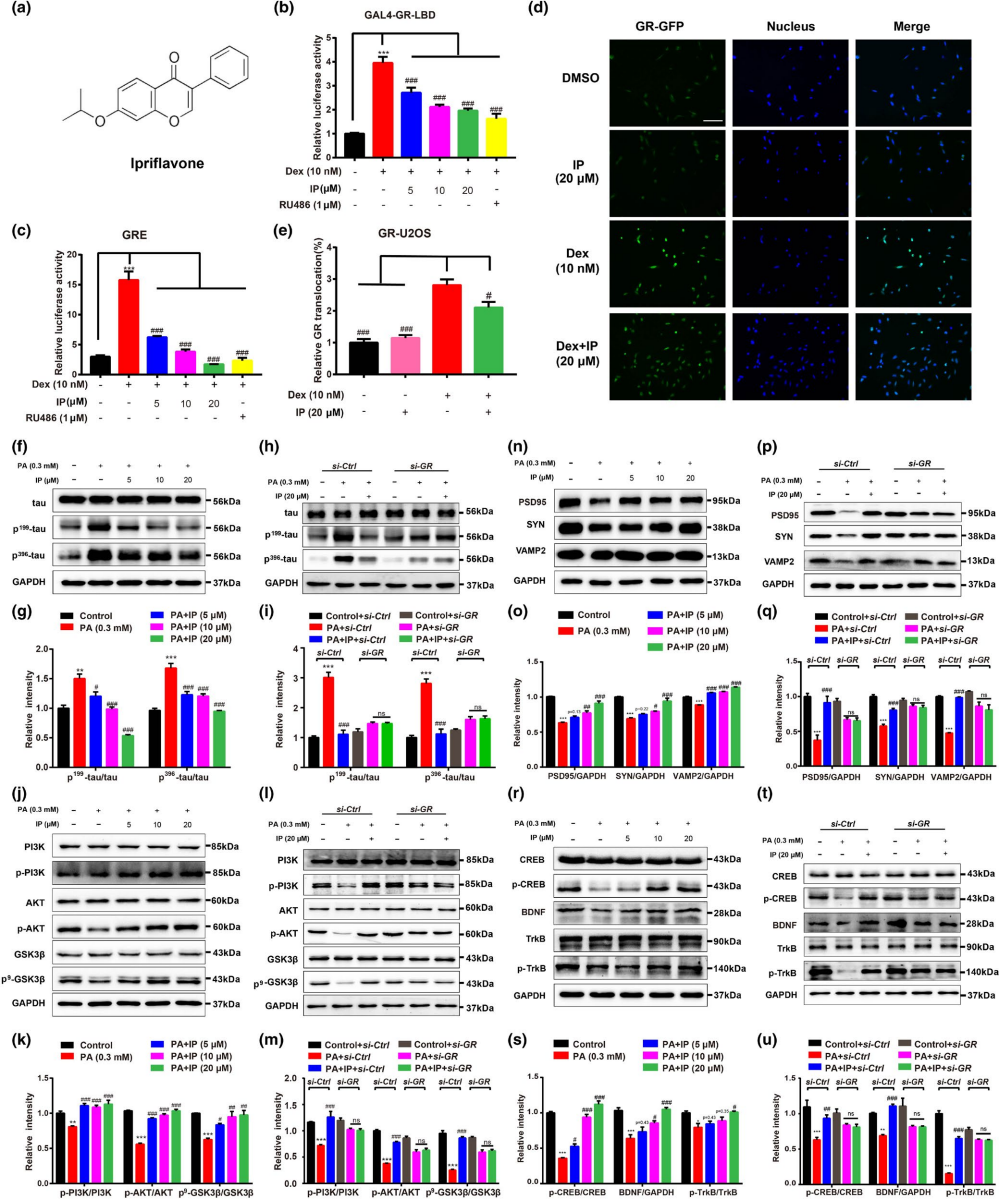
IP as a new non‐steroidal GR antagonist attenuated tau hyperphosphorylation through GR/PI3K/AKT/GSK3β pathway and ameliorated synaptic impairment through GR/CREB/BDNF/TrkB in primary neurons. (a) Chemical structure of IP. (b) Mammalian one‐hybrid and (c) transactivation assay results indicated that IP inhibited GR and antagonized the Dex‐induced GRE activity (*n* = 3). (d) Immunofluorescence assay and (e) its quantification (GFP fluorescence intensity ratio in nuclear and cytoplasm) results demonstrated that IP antagonized Dex‐stimulated GR nuclear translation (*n* = 6). Scale bar: 50 µm. (f‐i) Western blot and its quantification assays indicated that (f, g) IP antagonized PA‐induced tau hyperphosphorylation at Ser199 and Ser396 sites, and (h, i) *si*‐*GR* deprived IP of its antagonistic capability (*n* = 3). (j–m) Western blot and its quantification assays indicated that (j, k) IP antagonized PA‐induced decline in phosphorylation levels of PI3K, AKT (Ser 473) and GSK3β (Ser 9), and (l, m) *si*‐*GR* deprived IP of its antagonistic capability (*n* = 3). (n–q) Western blot assay with quantification results revealed that (n, o) IP antagonized PA‐induced suppression of synapse‐related proteins (PSD95, SYN, and VAMP2), and (p, q) *si*‐*GR* deprived IP of its antagonistic capability (*n* = 3). (r–u) Western blot assay with quantification results revealed that (r, s) IP antagonized PA‐induced suppression of protein levels of p‐CREB, BDNF, and p‐TrkB, and (t, u) *si*‐*GR* deprived IP of its antagonistic capability (*n* = 3). All assays were performed in primary neurons. GAPDH was used as loading control in Western blot assays. All values were presented as mean ± SEM. One‐way ANOVA and two‐way ANOVA followed by Dunnett's multiple comparison test. For Figure 1a‐e, *p < 0.05, **p < 0.01, ***p < 0.001 compared with DMSO group. ^#^ *p* < 0.05, ^##^ *p* < 0.01, ^###^ *p* < 0.001 compared with Dex group. For Figure 1f‐u, *p < 0.05, **p < 0.01, ***p < 0.001 compared with control or control +si‐*Ctrl* group. ^#^ *p* < 0.05, ^##^ *p* < 0.01, ^###^ *p* < 0.001 compared with PA or PA +si‐*Ctrl* group

Herein, we reported that ipriflavone (IP) (Figure 1a), a clinical drug for osteoporosis treatment, was determined to be a non‐steroidal GR antagonist. IP effectively improved the pathology of DCI in mice, and the underlying mechanism was intensively investigated by assay against the diabetic mice with GR‐specific knockdown in the brains by injection of adeno‐associated virus AAV‐ePHP‐*si*‐*GR*. To our knowledge, our work might be the first to expound the detailed mechanism underlying the regulation of GR against DCI‐like pathology and report the potential of IP in the treatment of DCI.

## 2 RESULTS

### 2.1 IP was a GR antagonist

*IP antagonized GR transactivation activity*—Given the efficiency and convenience in evaluation of nuclear receptor ligands for mammalian one‐hybrid accompanied with transactivation assays, both mammalian one‐hybrid and transactivation assays were here applied in HEK‐293T cells against the laboratory in‐house FDA‐approved drug library to find non‐steroidal GR antagonist. We determined that ipriflavone (No. 1012) displayed a high activity in antagonizing GR after screening total 2,268 reagents (part of the screening results was shown in Figure S1d–k).

As indicated in Figure 1b,c, both IP and GR antagonist Mife (as a positive control) antagonized the Dex (GR known agonist)‐activated reporter gene expression in mammalian one‐hybrid assay (Figure 1b) and antagonized the Dex‐stimulated luciferase gene expression in mammalian transactivation assay (Figure 1c). These results thus implied that IP was a potential antagonist of GR.

*IP suppressed Dex*‐*stimulated GR nuclear translocation*—GR as a transcription factor shuttles between cytosol and nucleus for regulating its downstream genes after stimulated by its agonist like Dex, and GR antagonist inhibits GR translocation (Q. Liu et al., 2010). With these facts, the potential effect of IP on GR cellular distribution was detected in U2OS/GR‐GFP stable cells. As indicated in Figure 1d,e, IP itself had no impacts on GR cellular distribution but antagonized the Dex‐stimulated GR nuclear translocation.

*IP was a selective antagonist of GR—*Given that GR as a member of nuclear receptor superfamily exhibits high homology with mineralocorticoid receptor (MR) (Weikum et al., 2017) and IP exhibits beneficial effects on postmenopausal osteoporosis (Gao et al., 2018) similar to estrogens that target estrogen receptors (ERs) (Geller et al., 1998), we evaluated the potential effects of IP on MR and ERs (ERα and ERβ).

Immunostaining assay result indicated that IP had no effects on MR nuclear translocation and failed to antagonize the CORT‐stimulated (CORT, corticosterone; a known MR agonist) MR nuclear translocation in HEK‐293T‐*MR*‐*GFP* cells (Figure S2a,b). In addition, mammalian transactivation assay (Liu et al., 2010) result also demonstrated that IP failed to antagonize the estrogen‐activated ERα/β reporter gene expression (Figure S2c).

Together, all results demonstrated that IP was a non‐steroidal GR antagonist.

### 2.2 IP attenuated tau hyperphosphorylation through GR/PI3K/AKT/GSK3β pathway in primary neurons

Given that tau hyperphosphorylation is responsible for the formation of neurofibrillary tangles (NFTs) that are highly attributable to DCI (Vossel et al., 2015), we investigated the potential of IP in ameliorating tau hyperphosphorylation in primary neurons.

*IP attenuated tau hyperphosphorylation by antagonizing GR*—In the assay, palmitic acid (PA) was used to induce diabetic pathology in primary neurons (Musi et al., 2018), and IP had no impacts on cell viability of primary neurons within the tested concentrations of 5–20 μM (Figure S2d). Western blot results indicated that IP antagonized the PA‐increased levels of phosphorylated‐tau at sites of Ser 396 and 199 (Figure 1f,g) and *si*‐*GR* deprived IP of its antagonistic capability (Figure 1h,i). These results thus demonstrated that IP suppressed tau hyperphosphorylation by inhibiting GR in primary neurons.

*IP attenuated tau hyperphosphorylation through GR*/*PI3K*/*AKT*/*GSK3β pathway in primary neurons*—Given that GSK3β as a kinase for tau hyperphosphorylation is activated in diabetic mice and regulated by PI3K/AKT signaling (Vossel et al., 2015), we inspected whether IP suppressed tau hyperphosphorylation through GR/PI3K/AKT/GSK3β pathway. Western blot results demonstrated that IP antagonized the PA‐decreased phosphorylation levels of PI3K, AKT, and GSK3β (Ser 9) (Figure 1j,k), and *si*‐*GR* deprived IP of its antagonistic capability (Figure 1l,m) in primary neurons.

Collectively, all results indicated that IP attenuated tau hyperphosphorylation through GR/PI3K/AKT/GSK3β pathway in primary neurons.

### 2.3 IP ameliorated synaptic impairment involving GR/CREB/BDNF/TrkB pathway in primary neurons

Given the close association of synapse plasticity and integrity with cognition (Batista et al., 2018), we assessed the potential protection of IP on synapse by Western blot assay in primary neurons.

*IP protected synaptic integrity*‐*related proteins by antagonizing GR—*As shown in Figure 1n,o, IP antagonized the PA‐induced decline in the protein levels of synaptic integrity‐related proteins PSD95, synaptophysin (SYN), and VAMP2, and *si*‐*GR* deprived IP of its antagonistic capabilities (Figure 1p,q).

*IP regulated CREB*/*BDNF*/*TrkB pathway by antagonizing GR—*By considering that brain‐derived neurotrophic factor (BDNF) as an important member of the neurotrophic factor family highly expresses in CNS (Egan et al., 2003) and CREB/BDNF/TrkB pathway functions potently in synaptic plasticity (Rauti et al., 2020), we investigated the potential regulation of IP against CREB/BDNF/TrkB pathway. Western blot results demonstrated that IP antagonized the PA‐repressed protein levels of p‐CREB, BDNF, and p‐TrkB (Figure 1r,s), and *si*‐*GR* deprived IP of its antagonistic abilities (Figure 1t,u).

Taken together, all results indicated that IP ameliorated synaptic impairment involving GR/CREB/BDNF/TrkB pathway in primary neurons.

### 2.4 IP suppressed inflammation through GR/NF‐κB/NLRP3/ASC/Caspase‐1 pathway in primary microglia

As indicated in the published reports, neuronal inflammation is tightly implicated in tau hyperphosphorylation, synaptic dysfunction, and cognitive impairment (Ndoja et al., 2020), while microglia are the central immune cells in CNS (Franco & Fernandez‐Suarez, 2015). With these facts, we inspected the potential of IP in alleviating neuroinflammation in primary microglia.

**FIGURE 2:**
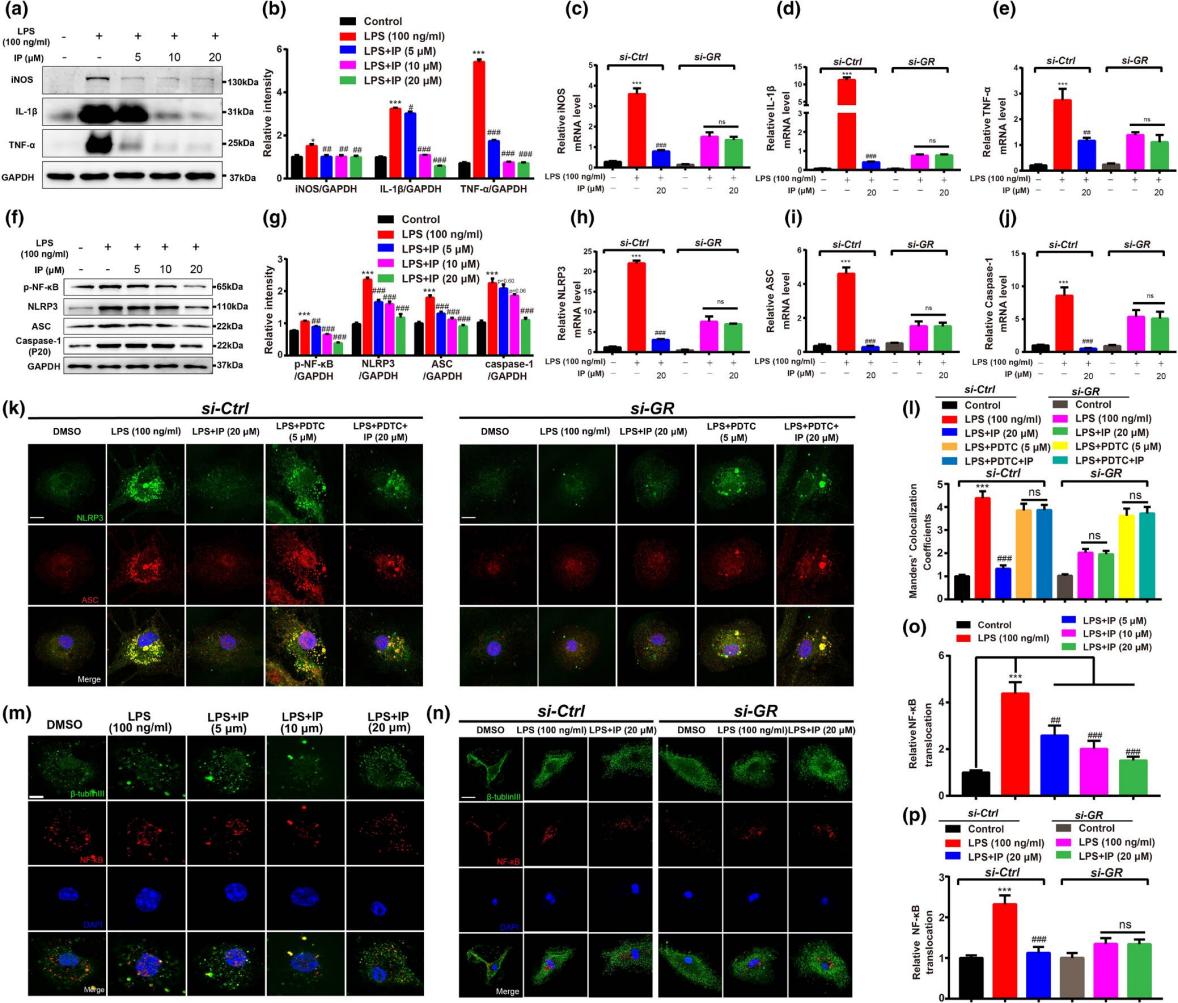
IP ameliorated inflammation through GR/NF‐κB/NLRP3/ASC/Caspase‐1 pathway in primary microglia. (a–j) Western blot and qPCR assays with quantification results revealed that (a, b) IP antagonized LPS‐stimulated protein levels of pro‐inflammatory factors iNOS, TNF‐α, and IL‐1β, and (c–e) *si*‐*GR* deprived IP of its antagonistic capability (*n* = 3); (f, g) IP antagonized LPS‐induced upregulation of p‐NF‐κB, NLRP3, ASC, and Caspase‐1(P20), and (h–j) *si*‐*GR* deprived IP of its antagonistic capability (*n* = 3). (k–p) Immunofluorescence assay with quantification results demonstrated that (k, l) treatment of NF‐κB inhibitor PDTC abolished the IP‐induced suppression against NLRP3; (m, o) IP suppressed NF‐κB nuclear translocation (*n* = 8; Scale bar: 5 µm), and (n, p) *si*‐*GR* deprived IP of its suppression capability (*n* = 6; Scale bar: 10 µm). All assays were performed in primary microglia. GAPDH was used as loading control in Western blot assays. All values were presented as mean ± SEM. One‐way ANOVA and two‐way ANOVA followed by Dunnett's multiple comparison test. *p < 0.05, **p < 0.01, ***p < 0.001 compared with control group. ^#^ *p* < 0.05, ^##^ *p* < 0.01, ^###^ *p* < 0.001 compared with LPS group

*IP suppressed inflammation by antagonizing GR—*Western blot and qPCR results revealed that IP antagonized the LPS‐induced increased pro‐inflammatory cytokines including iNOS, IL‐1β, and TNF‐α (Figure 2a,b and Figure S2g–i), while qPCR result demonstrated that *si*‐*GR* deprived IP of its above antagonistic capabilities (Figure 2c–e). Thus, IP suppressed inflammation by antagonizing GR.

*IP repressed NLRP3*/*ASC*/*Caspase*‐*1 pathway in primary microglia by antagonizing GR—*Given that NLRP3 as a potent intracellular pattern recognition receptor forms inflammasome with the adaptor molecule apoptosis‐associated speck‐like protein containing a CARD (ASC) and pro‐caspase‐1 leading to maturity and secretion of inflammatory cytokines IL‐1β and IL‐18 and further inflammation (Wang et al., 2018), we here carried out NLRP3‐related assays in primary microglia. Western blot results revealed that IP antagonized the LPS‐induced activation of protein levels of NLRP3, ASC, and Caspase‐1 (Figure 2f,g). Notably, qPCR and immunofluorescence assay results demonstrated that *si*‐*GR* deprived IP of its abovementioned antagonistic capabilities (Figure 2h–l). Additionally, immunofluorescence results also demonstrated that treatment of NF‐κB inhibitor PDTC blocked the capability of IP in suppressing NLRP3 expression (Figure 2k,l).

*IP restrained NF*‐*κB nuclear translocation in primary microglia by antagonizing GR—*By considering that NF‐κB as a key inflammation activator stimulates release of inflammatory cytokines (e.g., iNOS, TNF‐α) and NLRP3 inflammasome (Chen et al., 2020), we examined whether NF‐κB is essential for inflammation regulation in response to IP treatment in primary microglia. Western blot and immunofluorescence assay results demonstrated that IP antagonized the LPS‐stimulated protein levels of p‐NF‐κB (Figure 2f,g) and NF‐κB nuclear translocation (Figure 2m–o and Figure S2e,f), while *si*‐*GR* deprived IP of its antagonistic capabilities (Figure 2n–p). Thus, IP suppressed NF‐κB nuclear translocation by antagonizing GR.

All results indicated that IP suppressed NF‐κB/NLRP3/ASC/Caspase‐1 pathway by antagonizing GR.

### 2.5 IP improved cognitive impairment in diabetic mice by antagonizing GR

Next, we evaluated the capability of IP in ameliorating DCI in mice and investigated the related mechanisms by assay against the diabetic mice with GR‐specific knockdown in the brains by injection of AAV‐ePHP‐*si*‐*GR* (Chan et al., 2017).

The efficiency (around 50%) of GR knockdown in the brains by AAV‐ePHP‐*si*‐*GR* was detected after 2 weeks of injection of the virus (Figure S3a–j), and treatment of IP or AAV‐ePHP‐*si*‐*GR* had no impacts on blood glucose level, body weight, or food intake in diabetic mice (Figure S4a–l).

**FIGURE 3:**
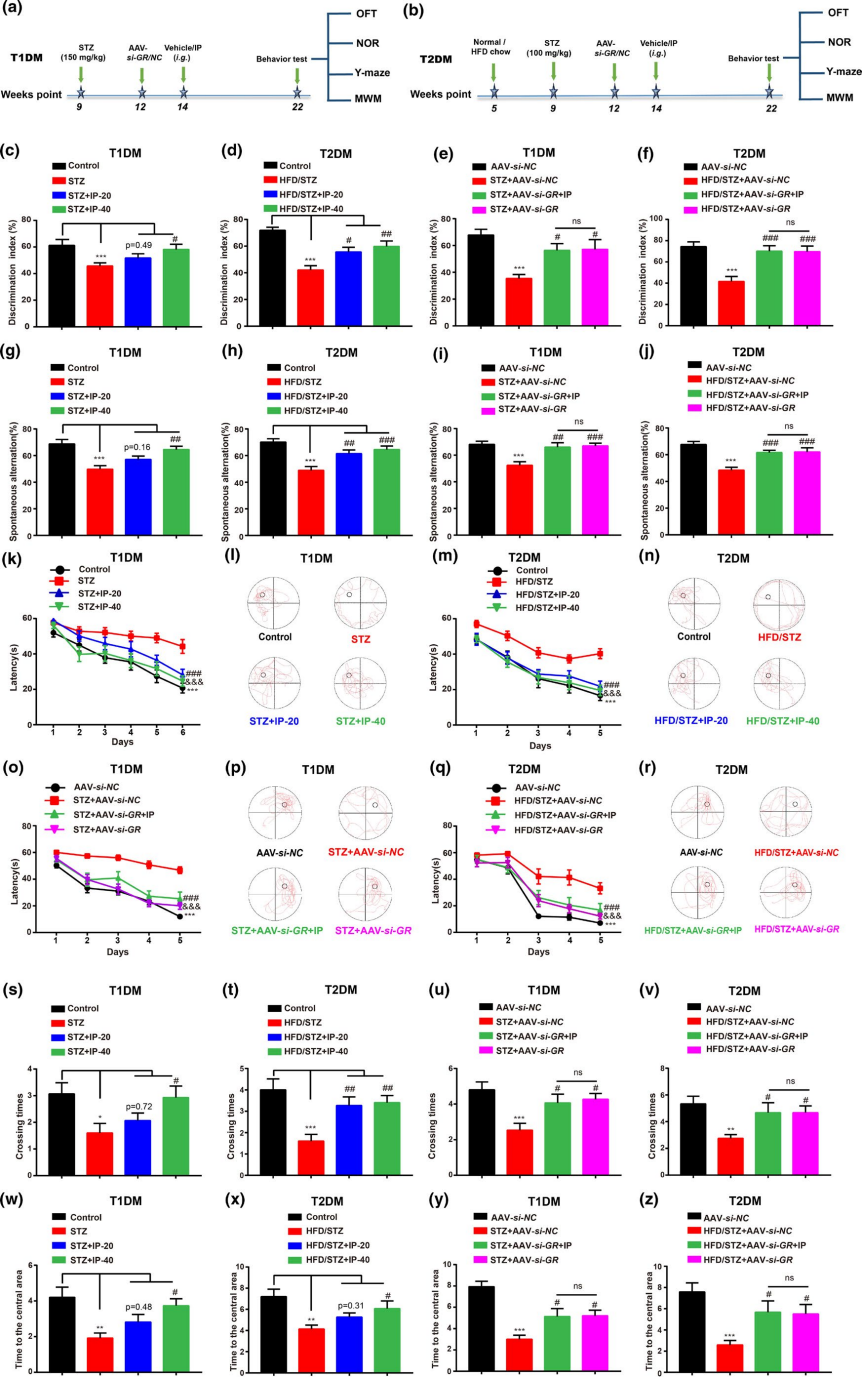
IP treatment ameliorated cognitive impairment in diabetic mice by antagonizing GR. (a, b) Experimental design of the study *in vivo*. (c–f) NOR test results indicated that IP (20, 40 mg/kg) or AAV‐*si*‐*GR* treatment ameliorated short‐term working memory defect in (c, e) STZ (*n* = 15 per group) and (d, f) HFD/STZ mice (*n* ≥ 10 per group), and IP (40 mg/kg) treatment failed to improve short‐term working memory defect in (e) STZ+AAV‐*si*‐*GR* (*n* = 15 per group) or (f) HFD/STZ+AAV‐*si*‐*GR* mice (*n* ≥ 10 per group). (g–j) Y‐maze test results indicated that IP (20, 40 mg/kg) or AAV‐*si*‐*GR* treatment ameliorated spatial working memory defect in (g, i) STZ (*n* = 15 per group) and (h, j) HFD/STZ mice (*n* ≥ 10 per group), and IP (40 mg/kg) failed to improve spatial working memory defect in (i) STZ+AAV‐*si*‐*GR* (*n* = 15 per group) or (j) HFD/STZ+AAV‐*si*‐*GR* diabetic mice (*n* ≥ 10 per group). (k, m, o, q) Escape latency results during platform trials in MWM test indicated that IP (20, 40 mg/kg) or AAV‐*si*‐*GR* treatment ameliorated learning and memory defects in (k, o) STZ (*n* = 15 per group) and (m, q) HFD/STZ mice (*n* ≥ 10 per group), and IP (40 mg/kg) failed to improve the defects in (o) STZ+AAV‐*si*‐*GR* (*n* = 15 per group) or (q) HFD/STZ+AAV‐*si*‐*GR* diabetic mice (*n* ≥ 10 per group). (l, n, p, r) Representative tracing graphs in escape latency trials for (l) STZ, (n) HFD/STZ, (p) STZ+AAV‐*si*‐*NC* and (r) HFD/STZ+AAV‐*si*‐*NC* diabetic mice. (s–v) Times of platform crossing in probe trials of MWM test indicated that IP (20, 40 mg/kg) or AAV‐*si*‐*GR* treatment ameliorated memory defects in (s, u) STZ (*n* = 15 per group) and (t, v) HFD/STZ mice (*n* ≥ 10 per group), and IP (40 mg/kg) failed to improve the defects in (u) STZ+AAV‐*si*‐*GR* (*n* = 15 per group) and (v) HFD/STZ+AAV‐*si*‐*GR* diabetic mice (*n* ≥ 10 per group). (w‐z) OFT result indicated that IP (20, 40 mg/kg) or AAV‐*si*‐*GR* treatment increased the exploring times of regional center in (w, y) STZ (*n* = 15 per group) and (x, z) HFD/STZ mice (*n* ≥ 10 per group), and IP (40 mg/kg) failed to perform such an improvement in (y) STZ+AAV‐*si*‐*GR* (*n* = 15 per group) or (z) HFD/STZ+AAV‐*si*‐*GR* diabetic mice (*n* ≥ 10 per group). All values were presented as mean ± SEM. One‐way ANOVA and two‐way ANOVA followed by Dunnett's multiple comparison test. *p < 0.05, **p < 0.01, ***p < 0.001 compared with control group or AAV‐*si*‐*NC* group. ^#^ *p* < 0.05, ^##^ *p* < 0.01, ^###^ *p* < 0.001 compared with T1DM (STZ; STZ+AAV‐*si*‐*NC*) or T2DM (HFD/STZ; HFD/STZ+AAV‐*si*‐*NC*) mice. ^&^ *p* < 0.05, ^&&^ *p* < 0.01 ^&&&^ *p* < 0.001 compared with T1DM (STZ; STZ+AAV‐*si*‐*NC*) or T2DM (HFD/STZ; HFD/STZ+AAV‐*si*‐*NC*) mice

*NOR test*—NOR test was performed to evaluate the short‐term working memory of mice. The results indicated that the discrimination indexes (DIs) in diabetic mice were expectedly decreased compared with those in control mice (Figure 3c–f). Treatment of IP or AAV‐*si*‐*GR* ameliorated DCI in diabetic mice (Figure 3c–f), and AAV‐*si*‐*GR* injection deprived IP of its ameliorative capability in AAV‐*si*‐*GR*‐injected diabetic mice (Figure 3e,f). Thus, these results suggested that IP improved short‐term working memory of diabetic mice by antagonizing GR.

*Y*‐*maze test*—Y‐maze test was used to evaluate the spatial working memory of mice. Treatment of IP or AAV‐*si*‐*GR* upregulated the rate of spontaneous alternation (Figure 3g–j) and the number of novel arm entrance (Figure S5a–d) in diabetic mice, and AAV‐*si*‐*GR* injection deprived IP of its capability in improving the abovementioned parameters in AAV‐*si*‐*GR*‐injected diabetic mice (Figure 3i,j and Figure S5c,d). The total distance had no significant difference among all groups (Figure S5e–h). Thus, all results implied that IP ameliorated spatial working memory of diabetic mice by antagonizing GR.

*MWM test*—MWM test was used to assess the effect of IP on spatial learning and long‐term memory (Achilly et al., 2021). As expected, diabetic mice spent more escape latency time and felt harder to find the location of platform precisely compared with control mice (Figure 3k–r), indicative of the dysfunction of learning and memory in diabetic mice. As indicated in Figure 3k–r and Figure S6a–d, treatment of IP or AAV‐*si*‐*GR* decreased the time of escape latency and improved the learning index (difference value of escape latency between two consecutive days) in diabetic mice. Additionally, the results of spatial probe test demonstrated that the crossing times and distance of the target quadrant (quadrant housing the platform) in diabetic mice were declined compared with those of control mice, and IP or AAV‐*si*‐*GR* treatment obviously rescued such a decline in diabetic mice (Figure 3s–v and Figure S6e–l). Notably, AAV‐*si*‐*GR* injection deprived IP of its ameliorative effects on latency, crossing times and distance in AAV‐*si*‐*GR*‐injected diabetic mice (Figure 3o–r,u,v and Figure S4c,d,g,h,k,l). Additionally, we did not observe a significant difference in total distance (Figure S4m–p) and speed (Figure S4q–t) among all groups. All results thus indicated that IP ameliorated spatial learning and long‐term memory dysfunction in diabetic mice by antagonizing GR.

*OFT test*—OFT test was employed to evaluate the beneficial effect of IP on exploratory behavior of diabetic mice. The results indicated that the exploring times and distances to the central area in diabetic mice were less than those in control mice, and treatment of IP or AAV‐*si*‐*GR* improved the abovementioned two indicators in diabetic mice (Figure 3w–z and Figure S7a–d). Notably, AAV‐*si*‐*GR* injection deprived IP of its beneficial effects on exploring times and distances in AAV‐*si*‐*GR* injected diabetic mice (Figure 3y,z and Figure S7c,d). The movement distance results of OFT showed no significant difference among all groups (Figure S7e–h). All results thus implied that IP improved exploratory behavior of diabetic mice by antagonizing GR.

In addition, IP had no effects on the behaviors of WT mice under the control conditions assayed by NOR (Figure S7i), Y‐maze (Figure S7j–l), MWM (Figure S7m–q), and OFT (Figure S7r–t) tests.

Taken together, all results indicated that IP treatment ameliorated cognitive impairment of diabetic mice by antagonizing GR.

### 2.6 IP attenuated tau hyperphosphorylation through GR/PI3K/AKT/GSK3β pathway in diabetic mice

**FIGURE 4:**
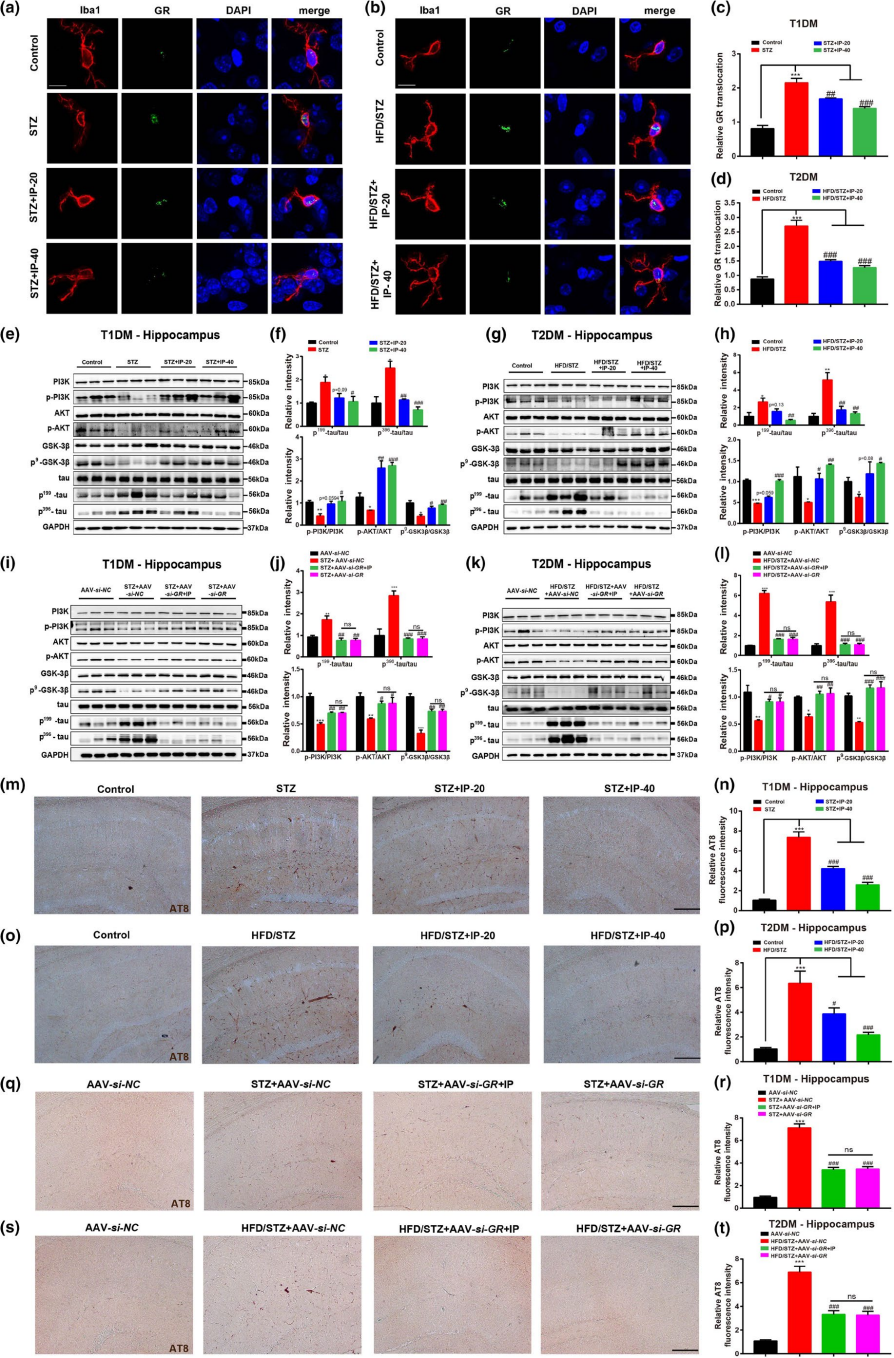
IP treatment attenuated tau hyperphosphorylation in diabetic mice through GR/PI3K/AKT/GSK3β pathway. (a–d) Immunofluorescence assay and its quantification results demonstrated that IP (20, 40 mg/kg) treatment suppressed GR nuclear translocation in hippocampal microglia of (a, c) STZ and (b, d) HFD/STZ mice (*n* = 6 per group). Scale bar: 10 µm. (e–l) Western blot and its quantification results demonstrated that (e, f) IP (20, 40 mg/kg) or AAV‐*si*‐*GR* treatment upregulated the protein levels of p‐PI3K, p‐AKT, and p^9^‐GSK3β, while downregulated p^199^‐tau and p^396^‐tau levels in hippocampus of (e, f, i, j) STZ and (g, h, k, l) HFD/STZ mice (*n* = 3 per group), and IP (40 mg/kg) treatment had no impacts on those proteins in (i, j) STZ+AAV‐*si*‐*GR* and (k, l) HFD/STZ+AAV‐*si*‐*GR* mice (*n* = 3 per group). (m–t) AT8 staining assay (PHF‐tau at sites of Ser 202 and Thr 205) and its quantification results demonstrated that IP (20, 40 mg/kg) or AAV‐*si*‐*GR* treatment repressed AT8 level in hippocampus of (m, n, q, r) STZ and (o, p, s, t) HFD/STZ mice, and IP (40 mg/kg) treatment had no impacts on AT8 level in (q, r) STZ+AAV‐*si*‐*GR* or (s, t) HFD/STZ+AAV‐*si*‐*GR* mice (AT8‐positive area was labeled brown, *n* = 6 per group). Scale bar: 100 µm. All values were presented as mean ± SEM. One‐way ANOVA followed by Dunnett's multiple comparison test. *p < 0.05, **p < 0.01, ***p < 0.001 compared with control group or AAV‐*si*‐*NC* group. ^#^ *p* < 0.05, ^##^ *p* < 0.01, ^###^ *p* < 0.001 compared with T1DM (STZ; STZ+AAV‐*si*‐*NC*) or T2DM (HFD/STZ; HFD/STZ+AAV‐*si*‐*NC*) mice

As we have determined the alleviation of IP on tau hyperphosphorylation in primary neurons (Figure 1f–i), we next investigated the potential of IP treatment in suppressing tau hyperphosphorylation in brain tissues of mice. Like the case in U2OS/GR‐GFP stable cell‐based assay (Figure 1d,e), we at first investigated whether IP treatment also inhibited GR nuclear translation in brains of mice. In the test, neurons and microglia in the hippocampus of diabetic mice were identified by MAP2 and Iba1 (He et al., 2021) antibodies, respectively, and the results indicated that IP inhibited GR nuclear translocation in microglia (Figure 4a–d) and neurons (Figure S8a–d). Meanwhile, the HPA axis related‐hormonal levels in serum of diabetic mice were detected by ELISA assay. As shown in Figure S8e–h, no significant difference was observed in level of corticosteroids (CORT) or corticotropin‐releasing hormone (CRH) among all groups, which was consistent with the previous reports (Asfeldt, 1972; Hackett et al., 2014). Thus, our results indicated that IP improved cognitive dysfunction in DCI mice through GR instead of HPA axis.

*IP attenuated tau hyperphosphorylation through GR*/*PI3K*/*AKT*/*GSK3β pathway in diabetic mice—*Next, we evaluated the potential of IP in suppressing tau hyperphosphorylation in diabetic mice. Western bolt results revealed that treatment of IP or AAV‐*si*‐*GR* upregulated p‐PI3K, p‐AKT, and p^9^‐GSK‐3β, and downregulated p^199^‐ and p^396^‐tau in the hippocampus (Figure 4e–l) and cortex (Figure S9a–l) of diabetic mice. Notably, AAV‐*si*‐*GR* injection deprived IP of its ability in suppressing tau hyperphosphorylation and PI3K/AKT/GSK3β pathway in AAV‐*si*‐*GR*‐injected diabetic mice (Figure 4i–l and Figure S9g–l).

In addition, AT8 staining and immunofluorescence results also demonstrated that treatment of IP or AAV‐*si*‐*GR* suppressed tau hyperphosphorylation (Ser 202, Thr 205 and Ser 396 site) in the hippocampus of diabetic mice (Figure 4m–t and Figure S9m–t), and AAV‐*si*‐*GR* injection deprived IP of its suppressive capability against tau hyperphosphorylation at these sites in the hippocampus of AAV‐*si*‐*GR*‐injected diabetic mice (Figure 4q–t and Figure S9q–t).

Collectively, all results implied that IP treatment attenuated tau hyperphosphorylation in diabetic mice through GR/PI3K/AKT/GSK3β pathway.

### 2.7 IP ameliorated synaptic impairment involving GR/CREB/BDNF/TrkB pathway in diabetic mice

**FIGURE 5:**
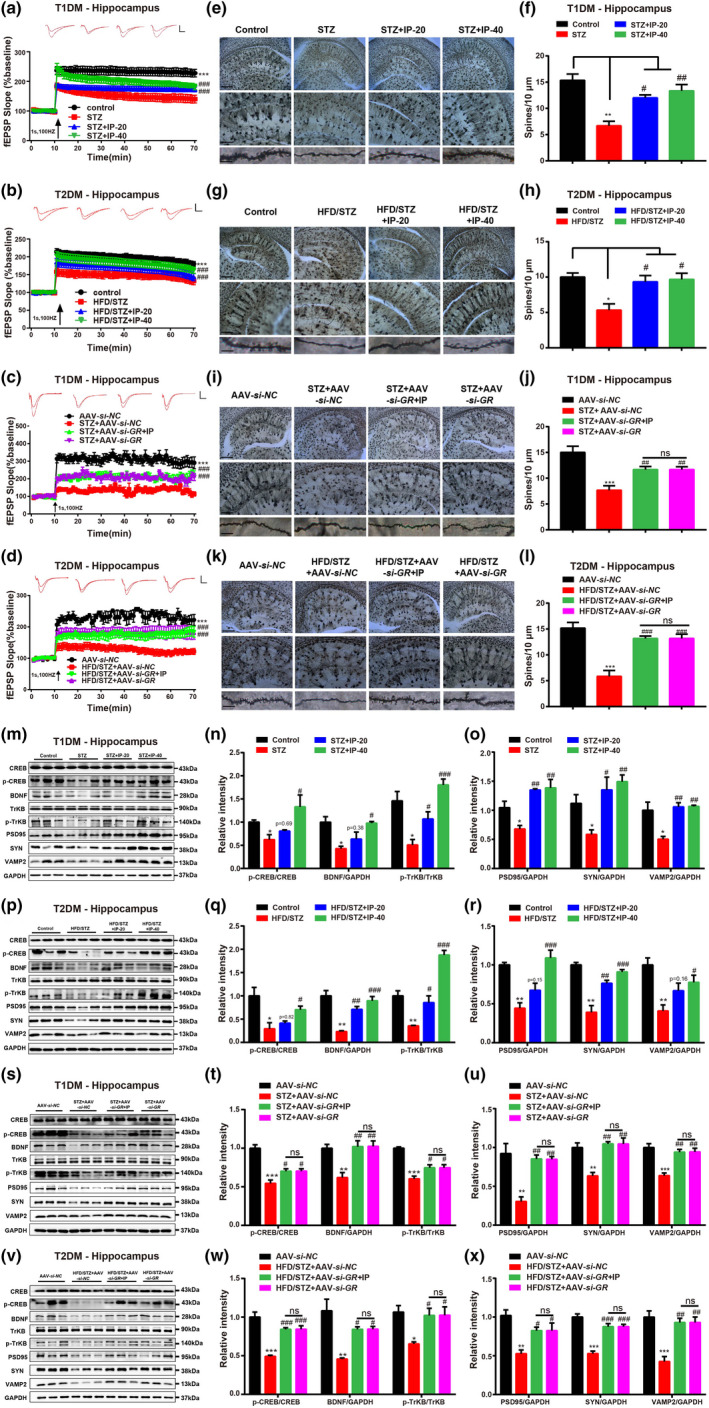
IP treatment protected synaptic impairment involving GR/CREB/BDNF/TrkB pathway in diabetic mice. (a–d) Electrophysiology results indicated that IP (20, 40 mg/kg) or AAV‐*si*‐*GR* treatment upregulated summary plots of mean normalized fEPSP slope after HFS (4 × 100 Hz) in hippocampus of (a, c) STZ and (b, d) HFD/STZ mice (*n* = 8 per group), while IP (40 mg/kg) treatment had no impacts on LTP impairment in (c) STZ+AAV‐*si*‐*GR* or (d) HFD/STZ+AAV‐*si*‐*GR* mice (*n* = 8 per group), representative sweep traces on the top. Scale bar: 1mv, 5ms. (e–l) Golgi‐Cox staining assay and its quantification results indicated that IP (20, 40 mg/kg) or AAV‐*si*‐*GR* treatment reversed spine density deficiency of hippocampal neuron in (e, f, i, j) STZ and (g, h, k, l) HFD/STZ mice, and IP (40 mg/kg) treatment had no impacts on such a deficiency in (i, j) STZ+AAV‐*si*‐*GR* or (k, l) HFD/STZ+AAV‐*si*‐*GR* mice (*n* = 6 per group). Scale bar: 200 µm, 100 µm, and 5µm, respectively. (m–x) Western blot and its quantification results demonstrated that IP (20, 40 mg/kg) or AAV‐*si*‐*GR* treatment upregulated the protein levels of p‐CREB, BDNF, p‐TrkB, PSD95, SYN, and VAMP2 in hippocampus of (m–o, s–u) STZ and (p–r, v–x) HFD/STZ (*n* = 3 per group), and IP (40 mg/kg) treatment had no impacts on these proteins in (s–u) STZ+AAV‐*si*‐*GR* or (v–x) HFD/STZ+AAV‐*si*‐*GR* mice (*n* = 3 per group). All values were presented as mean ± SEM. Two‐way ANOVA and one‐way ANOVA followed by Dunnett's multiple comparison test. *p < 0.05, **p < 0.01, ***p < 0.001 compared with control group or AAV‐*si*‐*NC* group. ^#^ *p* < 0.05, ^##^ *p* < 0.01, ^###^ *p* < 0.001 compared with T1DM (STZ; STZ+AAV‐*si*‐*NC*) or T2DM (HFD/STZ; HFD/STZ+AAV‐*si*‐*NC*) mice

*IP ameliorated long‐term potentiation (LTP) in diabetic mice by antagonizing GR—*Considering that long‐term potentiation (LTP) of synaptic transmission as a commonly experimental approach is widely used to assess synaptic plasticity and cognition related mechanism in mice (Kauer & Malenka, 2007), LTP‐related assay was here performed in the hippocampus of diabetic mice. As indicated in Figure 5a–d, fEPSP slopes in diabetic mice were decreased compared with those in control mice, and treatment of IP or AAV‐*si*‐*GR* effectively enhanced LTP in diabetic mice (Figure 5a–d). Notably, AAV‐*si*‐*GR* injection deprived IP of its abovementioned enhancement on LTP in AAV‐*si*‐*GR* injected diabetic mice (Figure 5c,d). Additionally, either single‐ or long‐term (2 weeks) administration of IP rendered no effects on LTP of WT mice under control conditions (Figure S10a,b). All results thus demonstrated that IP ameliorated LTP in diabetic mice by antagonizing GR.

*IP ameliorated synaptic integrity in diabetic mice by antagonizing GR—*Given that spine density is a key indicator of synaptic integrity (Leidig‐Bruckner & Ziegler, 2001), Golgi staining assay was next performed against the hippocampus of mice. As expected, diabetic mice exhibited a decreased spine density compared with control mice. It was noted that IP treatment or AAV‐*si*‐*GR* injection enhanced spine density in diabetic mice (Figure 5e–l), and AAV‐*si*‐*GR* injection deprived IP of its enhancive capability against spine density in AAV‐*si*‐*GR*‐injected diabetic mice (Figure 5i–l).

In addition, Western blot results also indicated that IP treatment or AAV‐*si*‐*GR* injection antagonized the downregulation of the synaptic integrity‐related proteins PSD95, SYN, and VAMP2 in the hippocampus (Figure 5m–x) and cortex (Figure S11a–l) of diabetic mice. Notably, AAV‐*si*‐*GR* injection deprived IP of its abovementioned antagonistic capability in AAV‐*si*‐*GR*‐injected diabetic mice (Figure 5s–x and Figure S11g–l). Thus, all results implied that IP protected synaptic integrity in diabetic mice by antagonizing GR.

*IP exhibited synaptic protection involving GR*/*CREB*/*BDNF*/*TrkB pathway in diabetic mice*—Like the case in cell‐based assays (Figure 1r–u), Western blot assay was also performed and the results demonstrated that treatment of IP or AAV‐*si*‐*GR* upregulated the protein levels of p‐CREB, BDNF, and p‐TrkB in the hippocampus (Figure 5m–w) and cortex (Figure S11a–k) of diabetic mice. Notably, AAV‐*si*‐*GR* injection deprived IP of its capability in regulating CREB/BDNF/TrkB pathway in AAV‐*si*‐*GR*‐injected diabetic mice (Figure 5s–w and Figure S11g–k).

Taken together, all results indicated that IP ameliorated synaptic impairment involving GR/CREB/BDNF/TrkB pathway in diabetic mice.

### 2.8 IP suppressed neuroinflammation in diabetic mice through GR/NF‐κB/NLRP3/ASC/Caspase‐1 pathway

**FIGURE 6:**
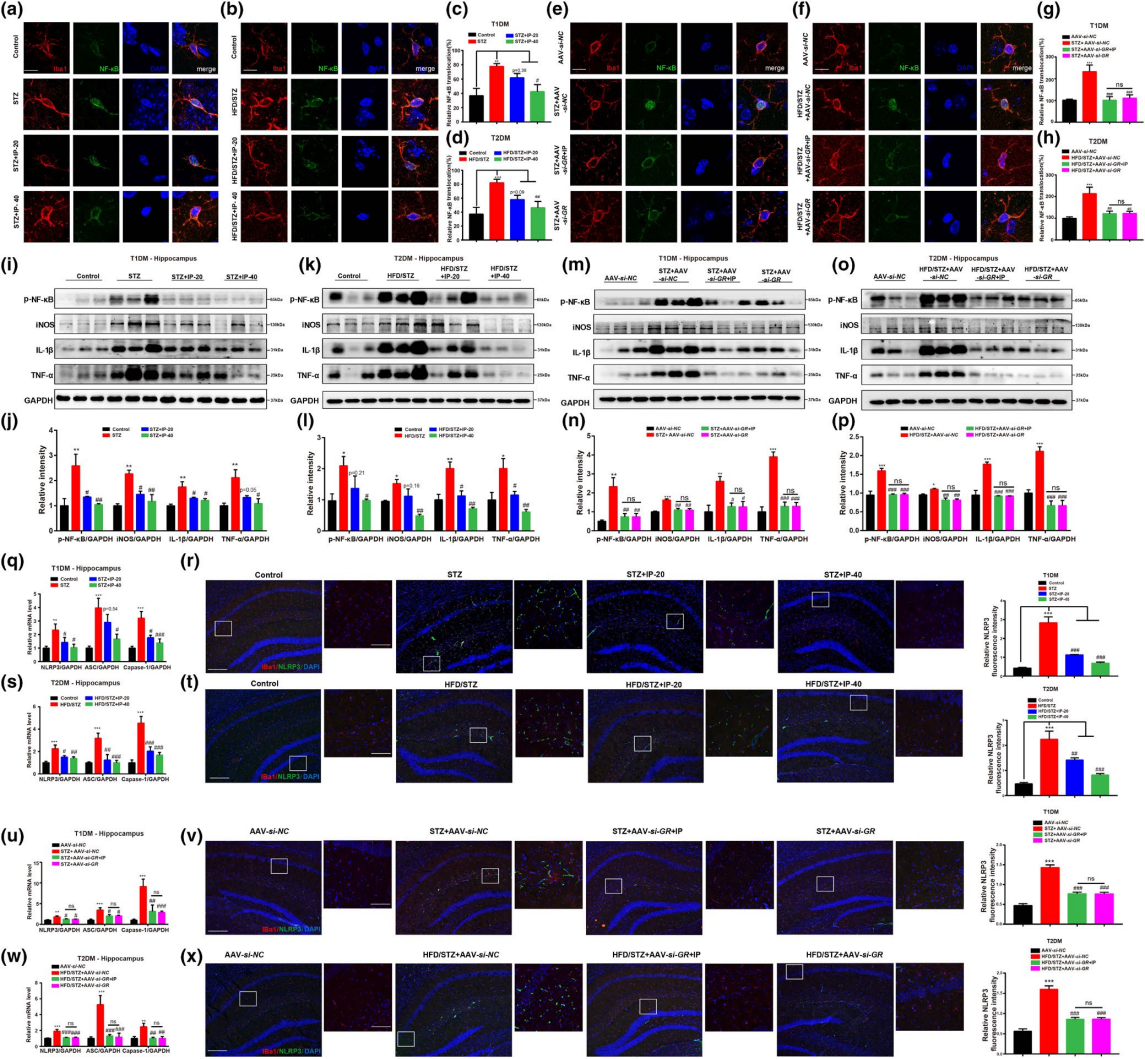
IP treatment repressed neuroinflammation through GR/NF‐κB/NLRP3/ASC/Caspase‐1 in diabetic mice. (a–h) Immunofluorescence assay and its quantification results demonstrated that IP (20, 40 mg/kg) or AAV‐*si*‐*GR* treatment suppressed the NF‐κB nuclear translocation in hippocampal microglia of (a, c, e, g) STZ and (b, d, f, h) HFD/STZ mice, and IP (40 mg/kg) treatment rendered no such effects in (e, g) STZ+AAV‐*si*‐*GR* and (f, h) HFD/STZ+AAV‐*si*‐*GR* mice (*n* = 6 per group). Scale bar: 10 µm. (i–p) Western blot and its quantification results indicated that IP (20, 40 mg/kg) or AAV‐*si*‐*GR* treatment repressed the protein levels of p‐NF‐κB, iNOS, TNF‐α, and IL‐1β in hippocampus of (i, j, m, n) STZ and (k, l, o, p) HFD/STZ mice, and IP (40 mg/kg) treatment rendered no such effects in (m, n) STZ+AAV‐*si*‐*GR* or (o, p) HFD/STZ+AAV‐*si*‐*GR* mice (*n* = 3 per group). (q, s, u, w) qPCR and (r, t, v, x) immunofluorescence assay results indicated that IP (20, 40 mg/kg) or AAV‐*si*‐*GR* treatment suppressed the levels of NLRP3, ASC, and Caspase‐1 in hippocampus of (q, r, u, v) STZ and (s, t, w, x) HFD/STZ mice, and IP (40 mg/kg) treatment had no such effects in (u, v) STZ+AAV‐*si*‐*GR* or (w, x) HFD/STZ+AAV‐*si*‐*GR* mice (*n* ≥ 6 per group). All values were presented as mean ± SEM. One‐way ANOVA followed by Dunnett's multiple comparison test. *p < 0.05, **p < 0.01, ***p < 0.001 compared with control group or AAV‐*si*‐*NC* group. ^#^ *p* < 0.05, ^##^ *p* < 0.01, ^###^ *p* < 0.001 compared with T1DM (STZ; STZ+AAV‐*si*‐*NC*) or T2DM (HFD/STZ; HFD/STZ+AAV‐*si*‐*NC*) mice

*IP restrained inflammation through GR*/*NF*‐*κB pathway in diabetic mice—*Immunofluorescence and Western blot assay results demonstrated that treatment of IP or AAV‐*si*‐*GR* suppressed NF‐κB nuclear translocation in hippocampal microglia (Figure 6a–h) and repressed the protein level of p‐NF‐κB in the hippocampus (Figure 6i–p) and cortex (Figures S12a–d and S13a–d) of diabetic mice. Additionally, Western blot and qPCR results also indicated that treatment of IP or AAV‐*si*‐*GR* effectively reduced the expressions of iNOS, IL‐1β, and TNF‐α in the hippocampus (Figure 6i–p; Figures S12k–p and S13k–p) and cortex (Figures S12a–j and S13a–j) of diabetic mice. Notably, AAV‐*si*‐*GR* injection deprived IP of its abovementioned repressive abilities (Figure 6e–h,m–p and Figure S13a–p). These results thus demonstrated that IP effectively repressed neuroinflammation through GR/NF‐κB signaling in diabetic mice.

*IP suppressed NLRP3 in diabetic mice by antagonizing GR—*qPCR and immunofluorescence assay results indicated that treatment of IP or AAV‐*si*‐*GR* inhibited the expressions of NLRP3, ASC, and caspase‐1 in the hippocampus (Figure 6q–x) and cortex (Figure S12q–v and Figure S13q–v) of diabetic mice, and AAV‐*si*‐*GR* injection deprived IP of its inhibitory ability against the abovementioned proteins (Figure 6s,t,w,x and Figure S13q–v) in AAV‐*si*‐*GR*‐injected diabetic mice. Thus, these results demonstrated that IP suppressed NLRP3/ASC/Caspase‐1 in diabetic mice by antagonizing GR.

Therefore, all results indicated that IP repressed neuroinflammation in diabetic mice through GR/NF‐κB/NLRP3 /ASC/Caspase‐1 pathway.

## 3 DISCUSSION

DCI is a common diabetic complication with complicated pathogenesis, and there has yet been no effective therapy against this disease (Biessels & Despa, 2018). Here, we determined that IP as a non‐steroidal GR antagonist efficiently improved DCI‐like pathology in mice and the underlying mechanisms have been intensively investigated. Our work has addressed the potency of GR antagonism in the amelioration of DCI and highlighted the potential of IP in treatment of this disease.

Tau hyperphosphorylation is widely acknowledged as the vital factor responsible for neuroinflammation and synapse loss in the pathology of DCI (Verma & Despa, 2019), and GSK3β as a key downstream signaling molecule of PI3K/AKT pathway is a potent kinase responsible for tau hyperphosphorylation (Jeong & Kang, 2018). In the pathology of DCI, tau hyperphosphorylation weakens the normal assembly function of microtubules and disrupts the transportation function of neuronal microtubule ultimately inducing neurotoxicity in neuronal cells (Kim et al., 2009). Notably, tau hyperphosphorylation induced by GSK3β could directly activate microglia followed by the release of pro‐inflammatory cytokines and finally the damage of the surrounding neurons (Biessels et al., 2006). Here, we determined the tight linkage of tau hyperphosphorylation to GR/PI3K/AKT/GSK3β signaling with IP as a probe. Our report may help better understand the regulation of GR in DCI and other tau hyperphosphorylation‐related diseases.

Neuroinflammation is closely associated with DCI pathology, and inflammation reduction promotes the recovery of synapse dysfunction and tauopathy (Ndoja et al., 2020). Microglia are the major macrophage in the central nervous system, and are responsible for regulation of neuron function and neuroinflammation (Keren‐Shaul et al., 2017), while the microglia‐mediated neuronal inflammation is closely associated with DCI pathology (McNeilly et al., 2016). NLRP3 inflammasome participates in the progression of synapse dysfunction and tau hyperphosphorylation (Denver et al., 2018). Pathologically, microglia are activated and NF‐κB may translocate from cytosol into nucleus regulating the downstream pro‐inflammatory cytokines such as TNF‐α and iNOS, which largely contributes to neurofibrillary tangles (NFTs) formation and synaptic dysfunction (Zhang et al., 2017). Here, we evidenced that IP suppressed inflammation through GR/NF‐κB/NLRP3/ASC/Capase‐1 pathway. To our knowledge, our work might be the first to report the mechanism underlying the anti‐inflammatory effect of GR antagonism in DCI‐like pathology involving NLRP3 inflammasome regulation, which may provide evidence for the potential of GR antagonism in the treatment of inflammation‐related diseases.

Notably, normal synaptic function is essential for learning and memory, and its deficiency aggravates DCI progression (Kauer & Malenka, 2007). In DCI pathology, neuronal inflammation and tau hyperphosphorylation exacerbate synapse damage substantially triggering the impairment of learning and memory (Verma & Despa, 2019). Tau hyperphosphorylation leads to destabilization of microtubules and interruption of axonal transport, which are associated with synaptic dysfunction (Verma & Despa, 2019), while neuronal inflammation hinders neurite formation and damages axonal myelination thus weakening synchronized synaptic activity. In that case, tau hyperphosphorylation and inflammation synergistically impair synaptic function (Biessels et al., 2006). BDNF is an important member of the neurotrophic factor family and highly expressed in the central nervous system (Parkhurst et al., 2013). It plays a potent role in learning and memory via enhancing the synapse plasticity, altering the morphology of neurons, increasing the density of synaptic terminals, and promoting the growth of dendrites and axons (Afonso et al., 2019). CREB as a cellular transcription factor functions potently in the maintenance of synaptic plasticity and integrity (Barco et al., 2002). Here, we found that IP improved synaptic impairment involving GR/CREB/BDNF/TrkB pathway. Our results have disclosed the mechanism underlying the improvement of GR antagonism against synaptic deficit.

IP is a natural isoflavone derivative being clinically used for osteoporosis treatment (Makita & Ohta, 2002). Notably, diabetes is a predisposing factor of osteoporosis, and patients with diabetes were reported to have a higher risk of fracture, in that the level of insulin‐like growth factor 1 (IGF‐1), a marker of bone formation, is lower in patients with insulin‐dependent diabetes than healthy individuals (Leidig‐Bruckner & Ziegler, 2001). It was reported that glucocorticoids imbalance may induce osteoporosis by GR‐dependent or GR‐independent pathway (Hua et al., 2019). Here, our report that IP was a non‐steroidal GR antagonist has revealed the potent target information for IP in the clinical treatment of osteoporosis.

## 4 CONCLUSIONS

In conclusion, IP as a non‐steroidal GR antagonist effectively ameliorates DCI in mice. The underlying mechanism has been intensively investigated, in that IP suppressed tau hyperphosphorylation through GR/PI3K/AKT/GSK3β pathway, alleviated neuronal inflammation through GR/NF‐κB/NLRP3/ASC/Caspase‐1 pathway, and improved synaptic impairment involving GR/CREB/BDNF pathway. Collectively, our work has highly addressed the potency of non‐steroidal GR antagonist in treating DCI and highlighted the potential of IP in the treatment of this disease.

## 5 EXPERIMENTAL PROCEDURES

### 5.1 Study design

Non‐steroidal GR inhibitor IP was screened from laboratory in‐house FDA‐approved drug library through mammalian one‐hybrid and transactivation assays in HEK‐293T cells. The potential of IP in the amelioration of DCI‐like pathology were evaluated by a series of cell‐based assays (tau pathology, synaptic integrity, and neuroinflammation) and related behaviors tests (Morris water maze, Y‐maze, New object recognition, Open field test) against STZ‐induced diabetic mice. The mechanism underlying the amelioration of IP on DCI‐like pathology was investigated by assay against diabetic mice with GR knockdown in the brain by injection of AAV‐ePHP‐*si*‐*GR*.

For all animal studies, mice were litter‐matched, age‐matched, and gender‐matched to keep all data agree with each other. Completely random grouping design and exploratory experimental research were performed based on the experimental animals.

Investigators who conducted the experiments or analyzed the data were blinded to group.

### 5.2 Statistical analysis

All data were expressed as mean ± SEM. Statistical analysis was performed by using GraphPad Prism 7.0. One‐way ANOVA and two‐way ANOVA with Dunnett's post‐test were performed to analyze the significant difference between multiple treatments and the control. *p* < 0.05 was considered as statistically significant (one‐way ANOVA test in cellular assays; two‐way ANOVA test in *siRNA* interference assays, escape latency of MWM tests and LTP assays; one‐way ANOVA test in animal assays).

## CONFLICT OF INTEREST

The authors declare that they have no conflict of interest. All institutional and national guidelines for the care and use of laboratory animals were followed.

## AUTHOR CONTRIBUTIONS

X.S. and R.N. designed the study. X.S. reviewed the manuscript. R.N performed research; R.N. performed the animal and cell experiments. R.N. analyzed and interpreted data. X.S., X.O., D.Z., R.X., Y.H., T.Z., X.Z., and Y.L. helped the animal experiments. R.N., J.L., M.Q., and J.W. wrote the manuscript. R.N., X.S., J.W., J.L., and M.Q. are the guarantors of this work and, as such, have full access to all data in the study and take responsibility for the integrity of the data and the accuracy of the data analysis. All authors approved the manuscript.

## Supporting information

Fig S1

Fig S2

Fig S3

Fig S4

Fig S5

Fig S6

Fig S7

Fig S8

Fig S9

Fig S10

Fig S11

Fig S12

Fig S13

Supplementary Material

## Data Availability

The data sets used and/or analyzed during the current study are available from the corresponding author on reasonable request.
