# Supplementary figures and images for: Ipriflavone as a non‐steroidal glucocorticoid receptor antagonist ameliorates diabetic cognitive impairment in mice
Source: Aging Cell. 2022 Feb 16;21(3):e13572. doi: 10.1111/acel.13572 (PMC8920458; doi:10.1111/acel.13572)

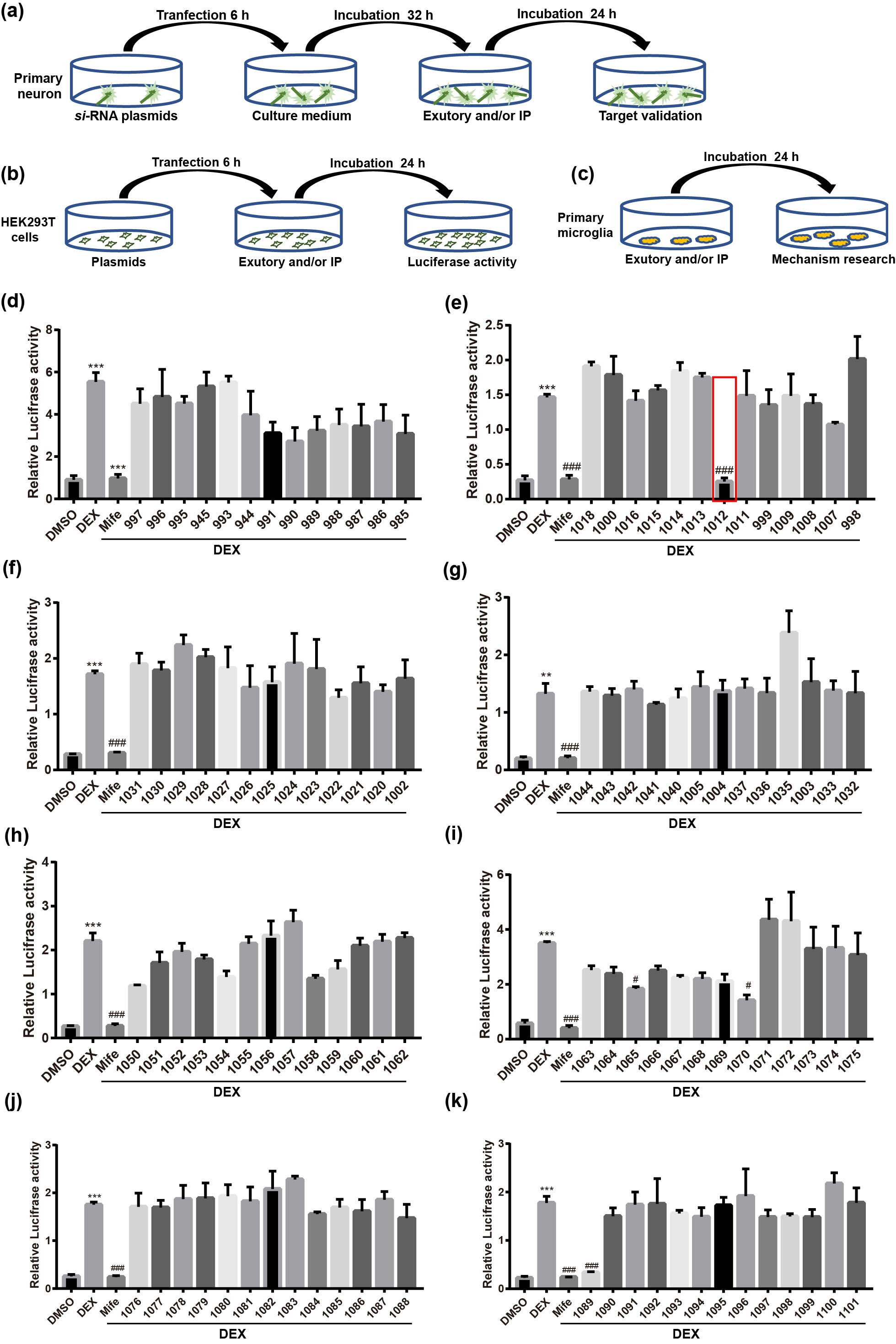

Supplement: Supplementary file 1 — Fig S1 [file ACEL-21-e13572-s005.jpg]

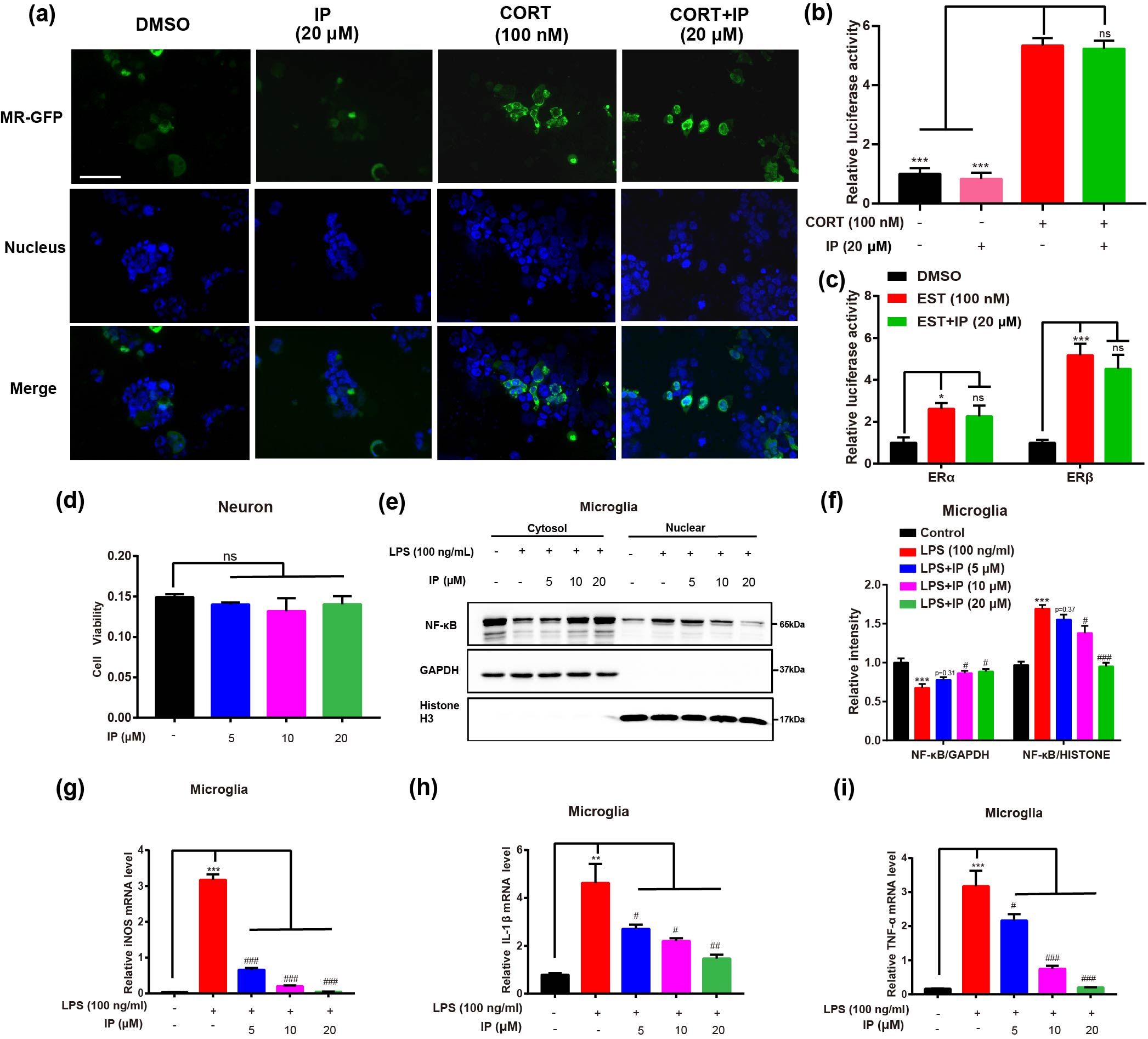

Supplement: Supplementary file 2 — Fig S2 [file ACEL-21-e13572-s004.jpg]

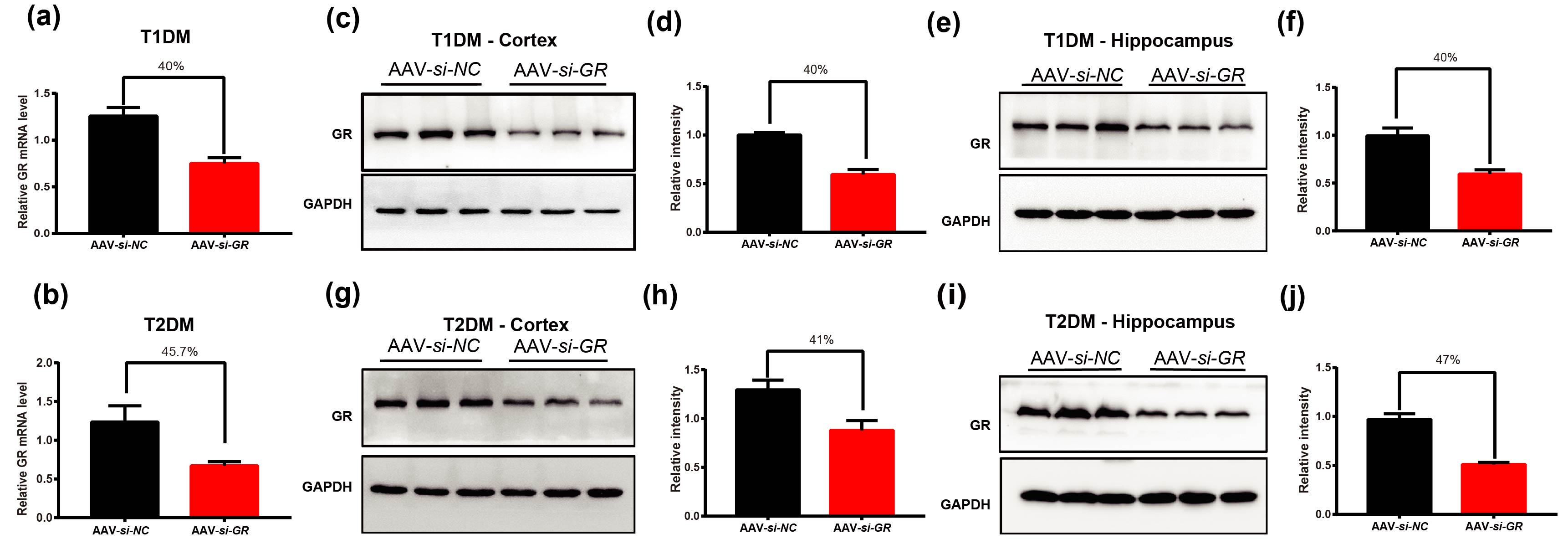

Supplement: Supplementary file 3 — Fig S3 [file ACEL-21-e13572-s008.jpg]

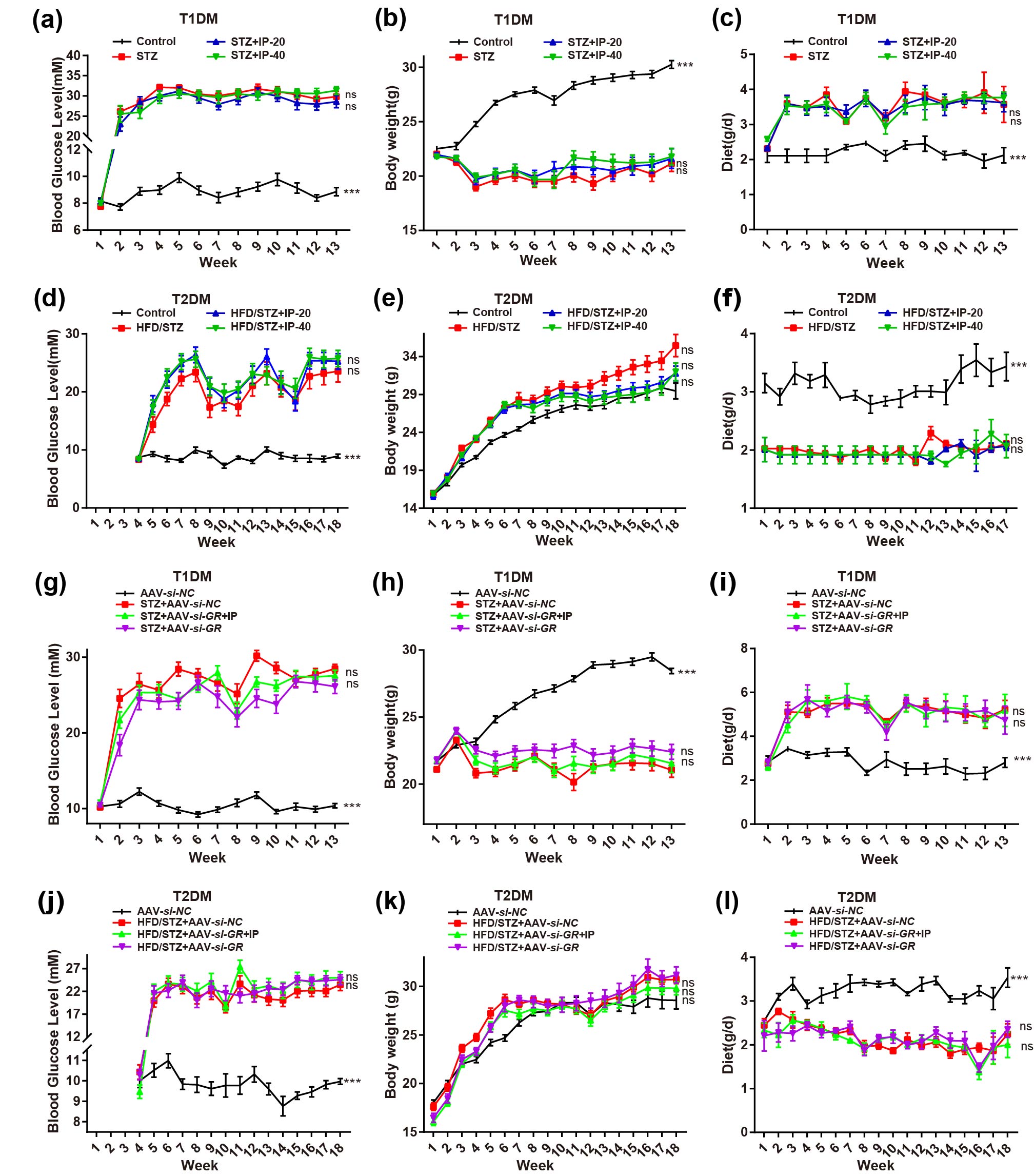

Supplement: Supplementary file 4 — Fig S4 [file ACEL-21-e13572-s002.jpg]

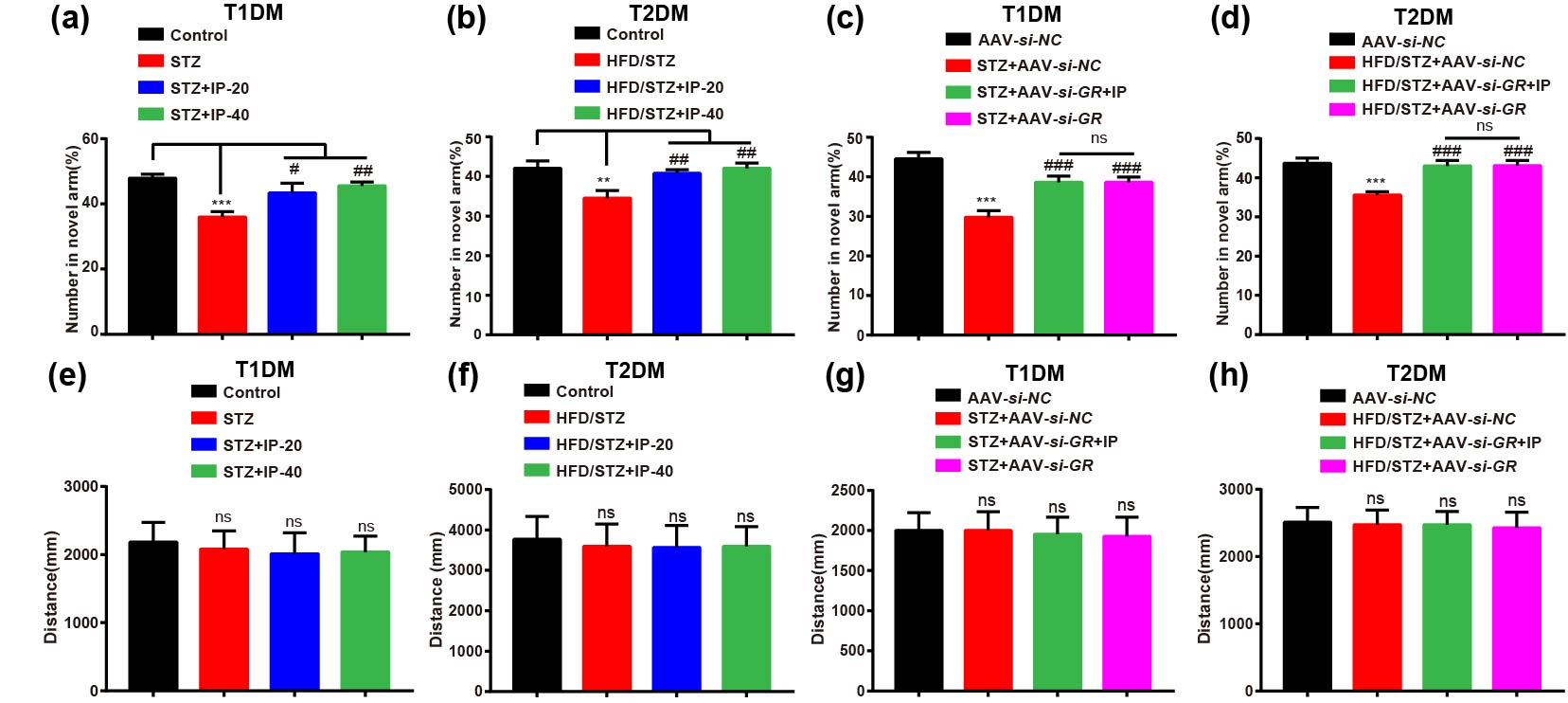

Supplement: Supplementary file 5 — Fig S5 [file ACEL-21-e13572-s014.jpg]

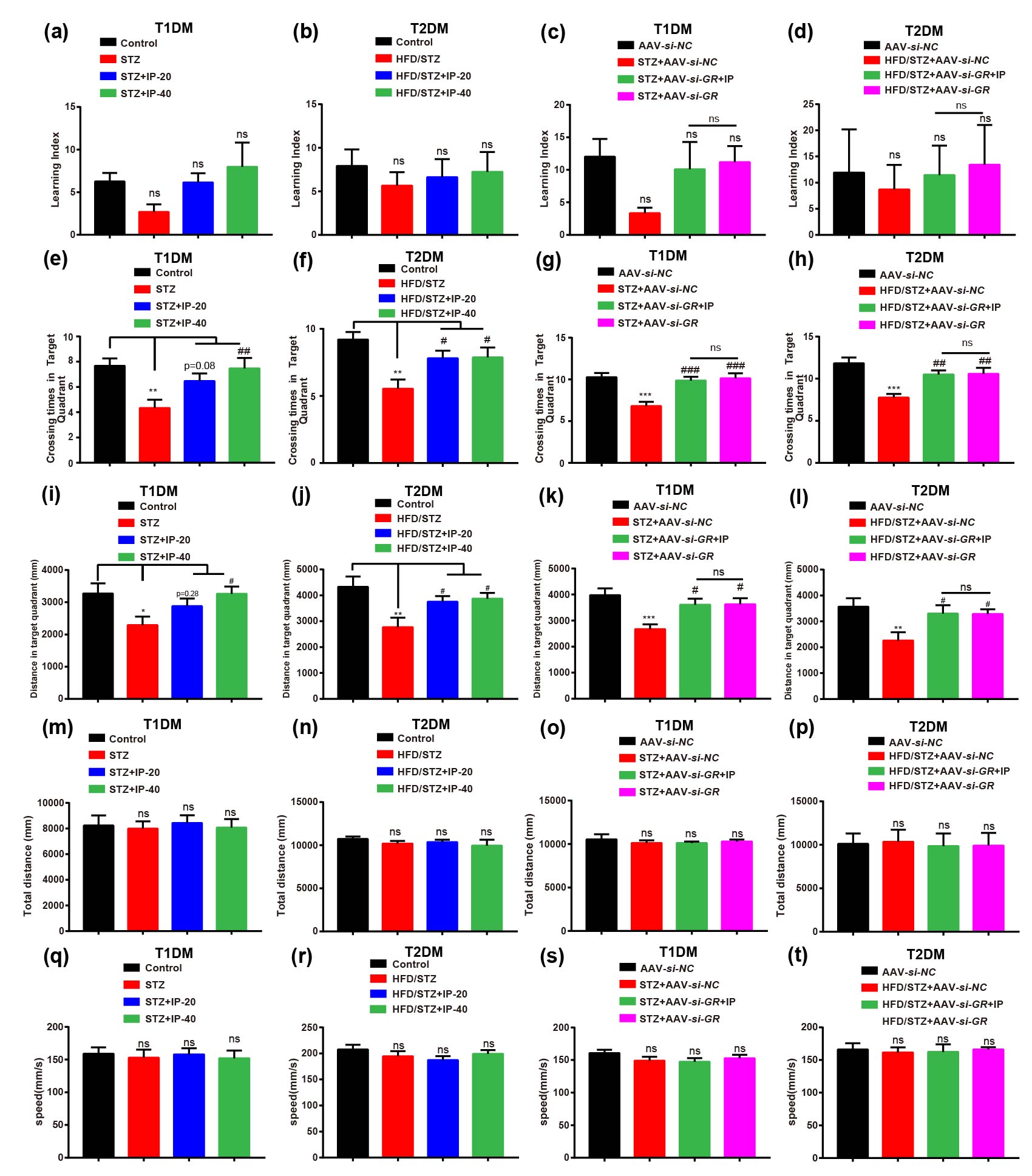

Supplement: Supplementary file 6 — Fig S6 [file ACEL-21-e13572-s010.jpg]

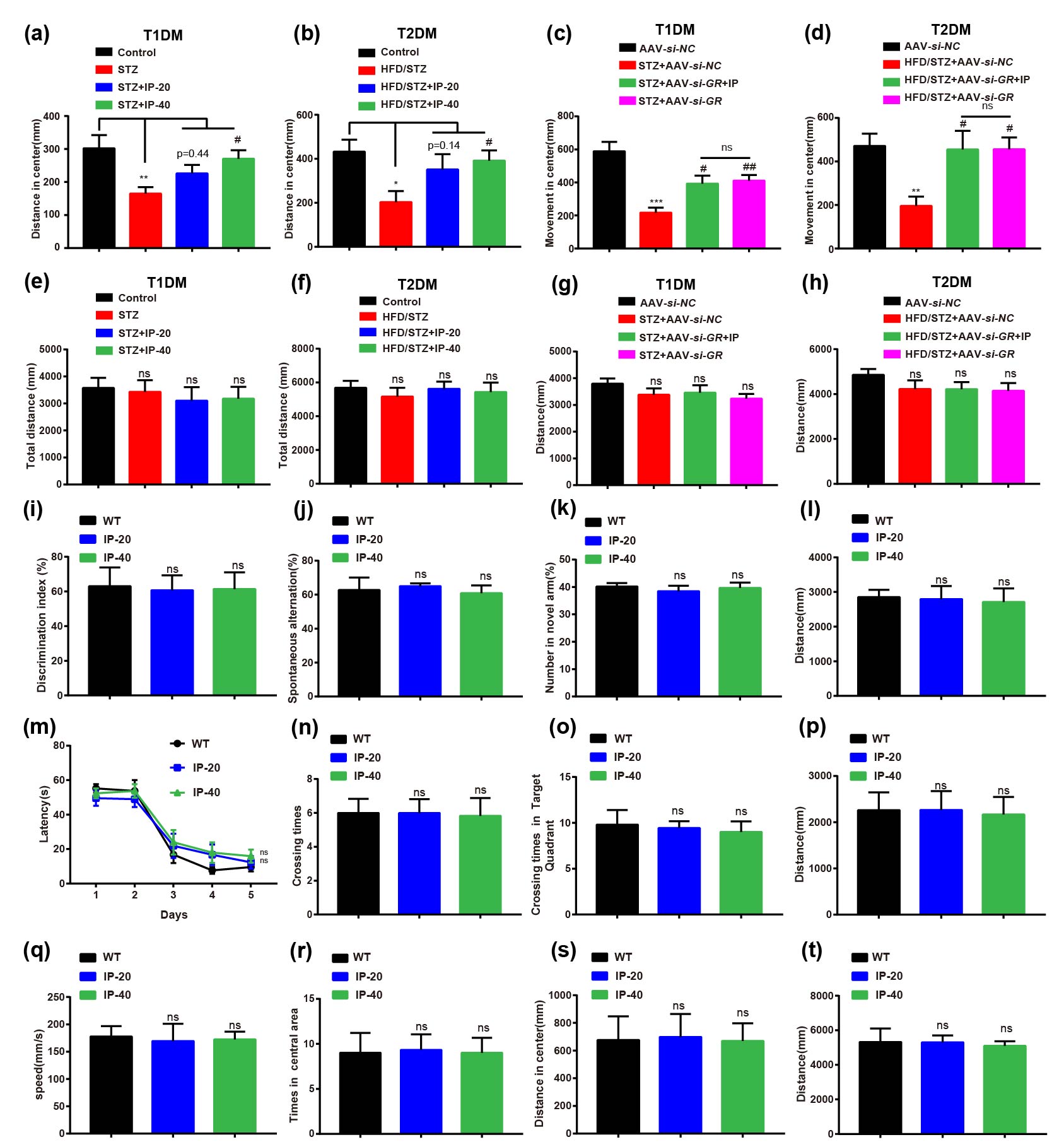

Supplement: Supplementary file 7 — Fig S7 [file ACEL-21-e13572-s001.jpg]

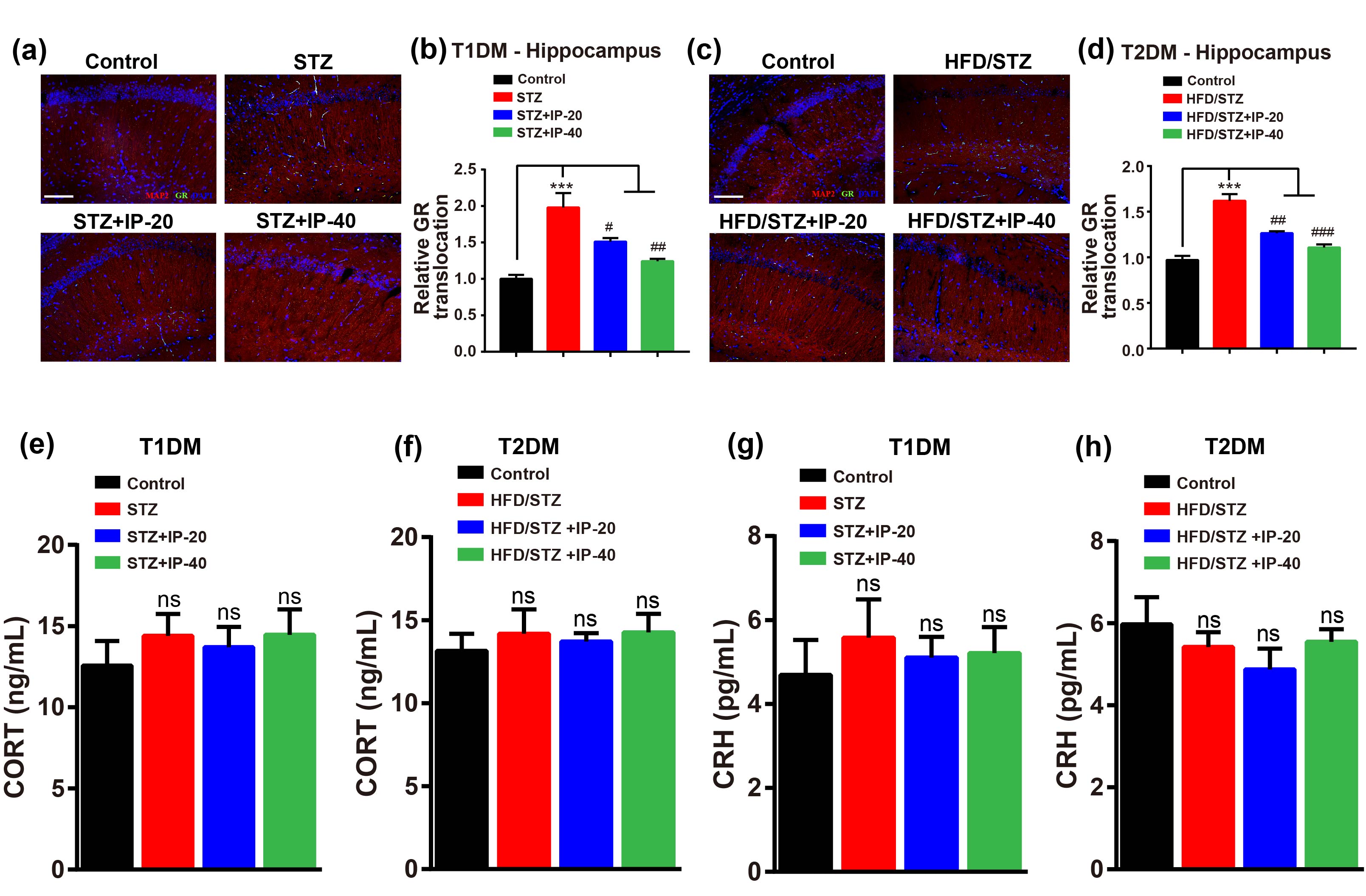

Supplement: Supplementary file 8 — Fig S8 [file ACEL-21-e13572-s013.jpg]

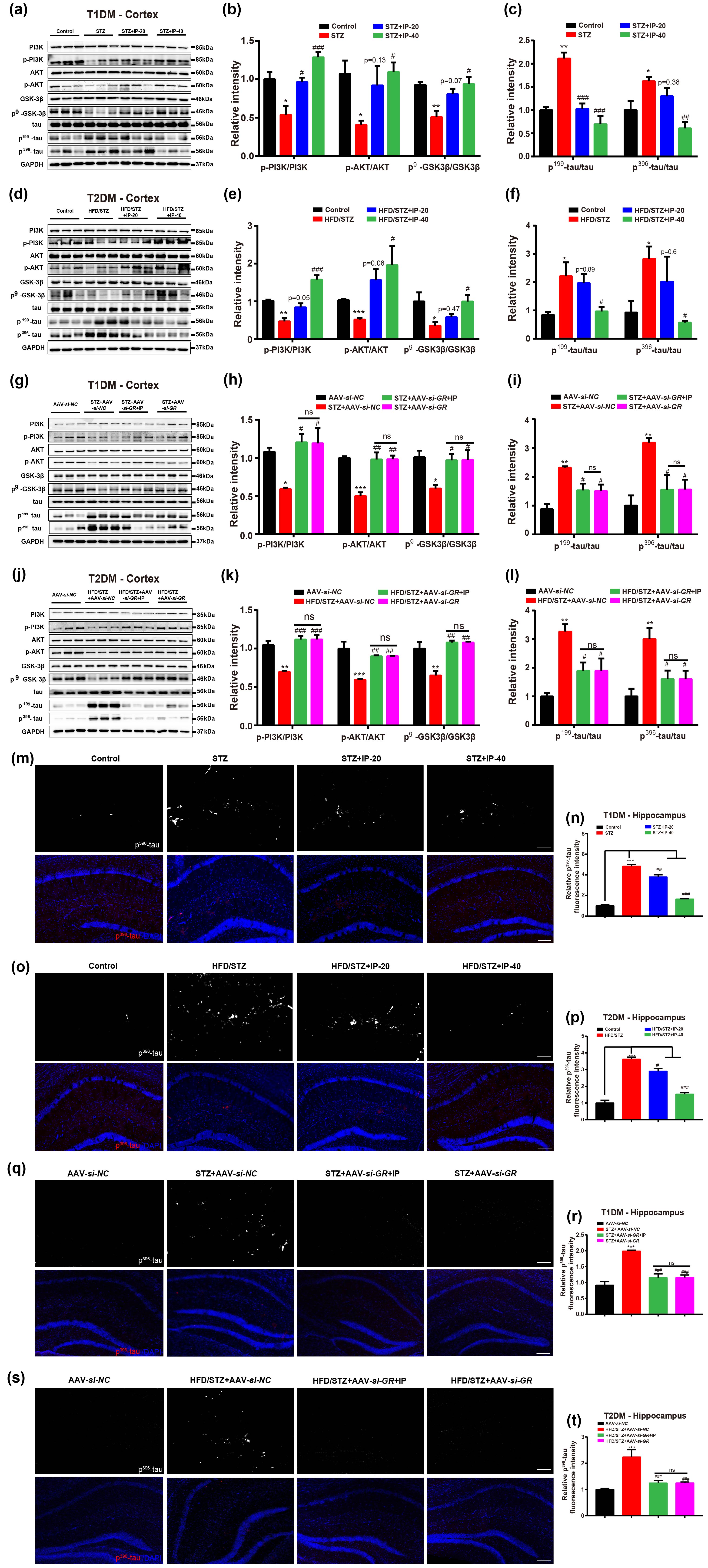

Supplement: Supplementary file 9 — Fig S9 [file ACEL-21-e13572-s011.jpg]

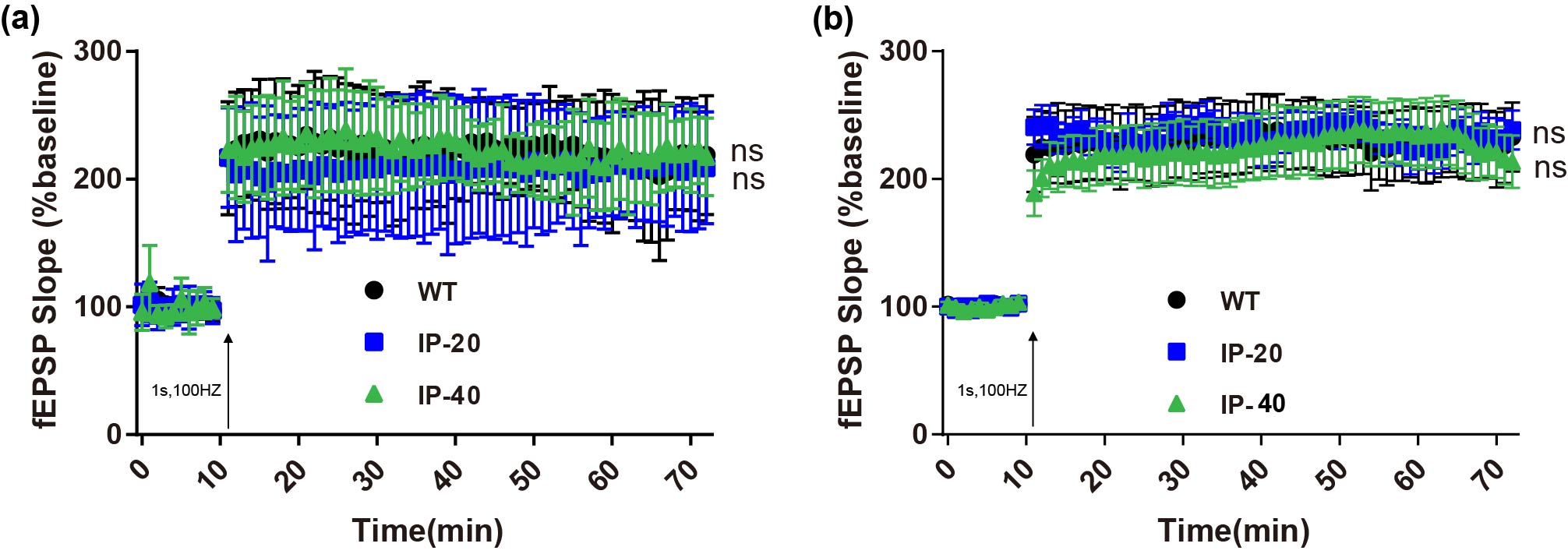

Supplement: Supplementary file 10 — Fig S10 [file ACEL-21-e13572-s003.jpg]

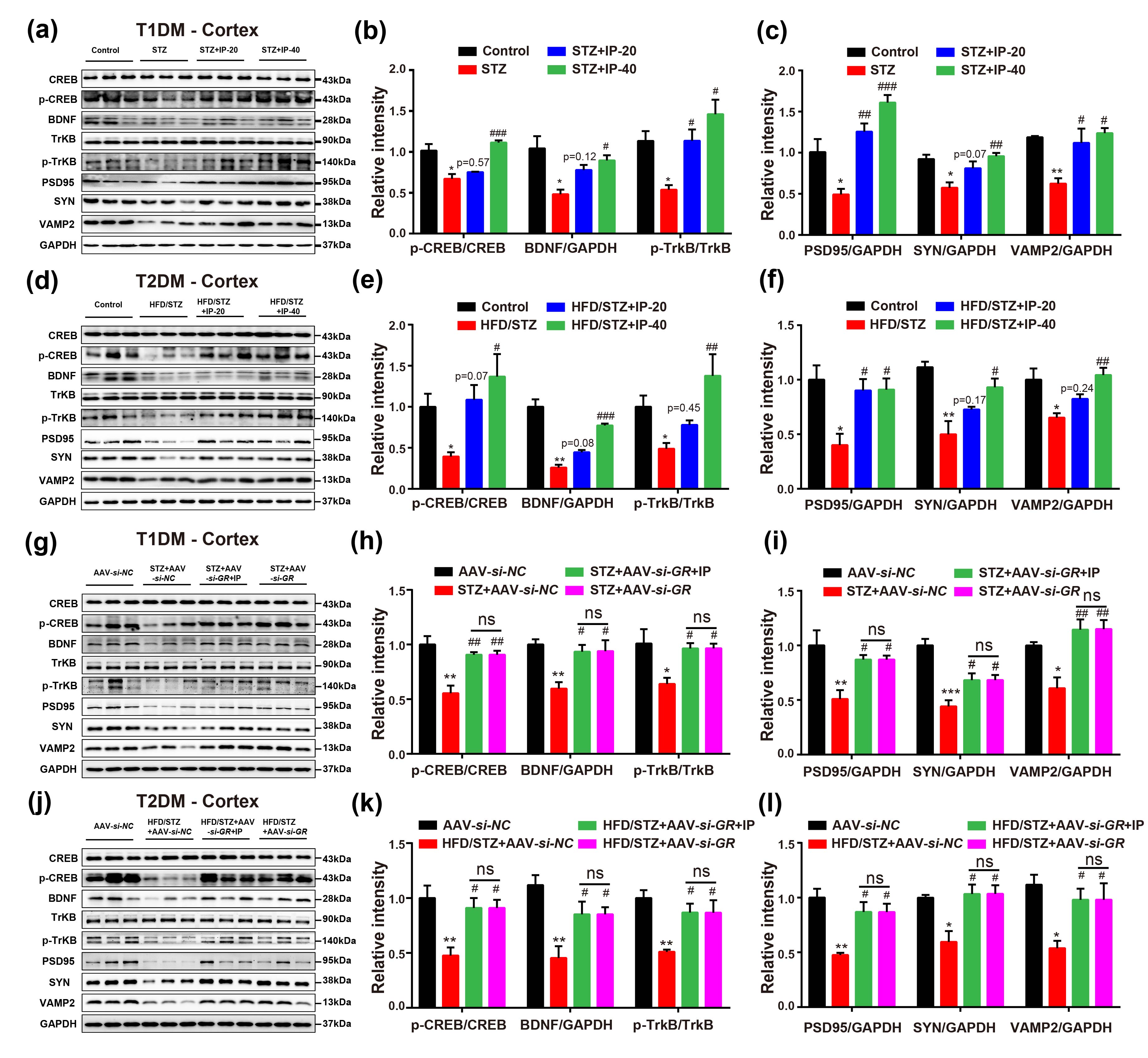

Supplement: Supplementary file 11 — Fig S11 [file ACEL-21-e13572-s006.jpg]

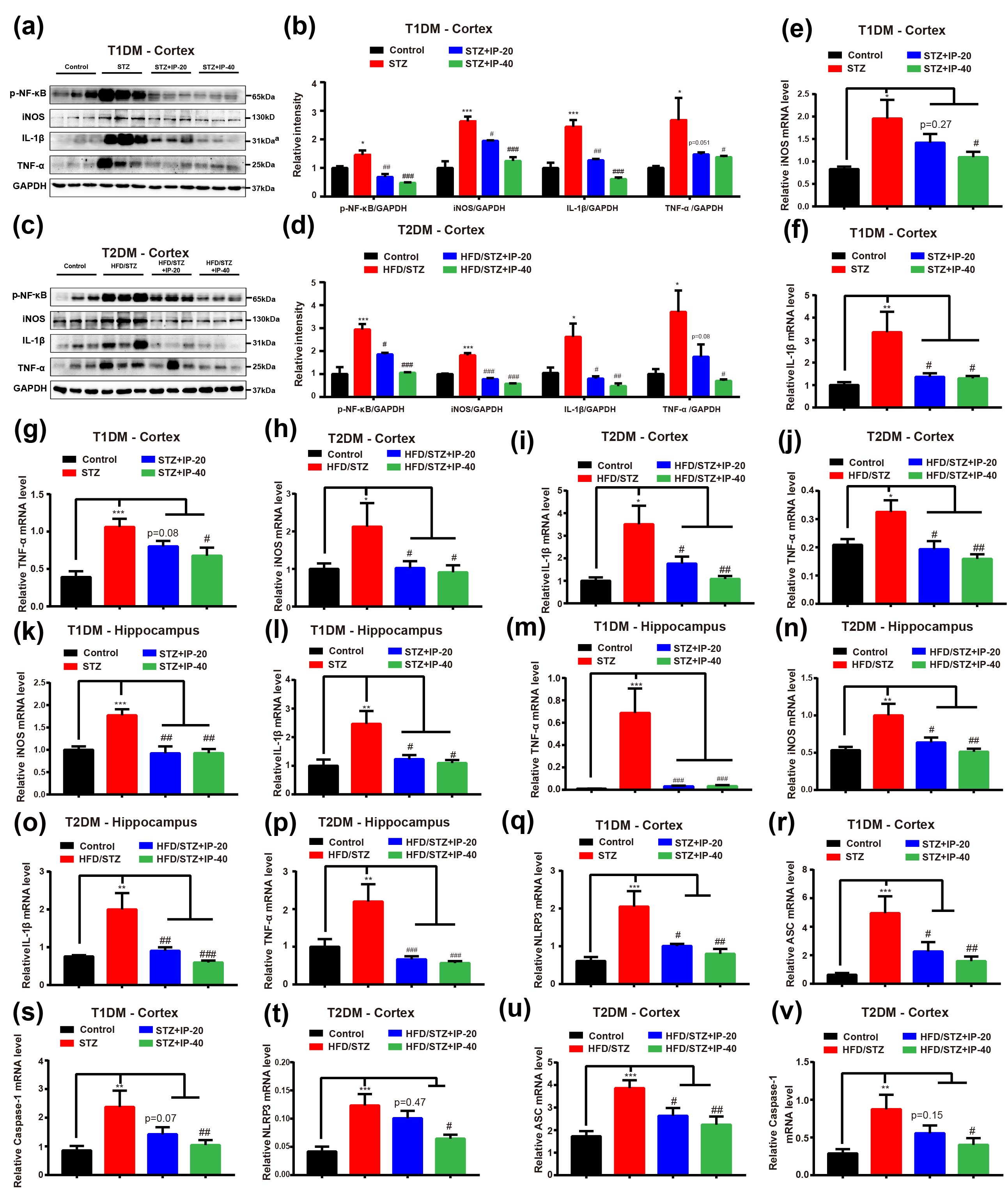

Supplement: Supplementary file 12 — Fig S12 [file ACEL-21-e13572-s007.jpg]

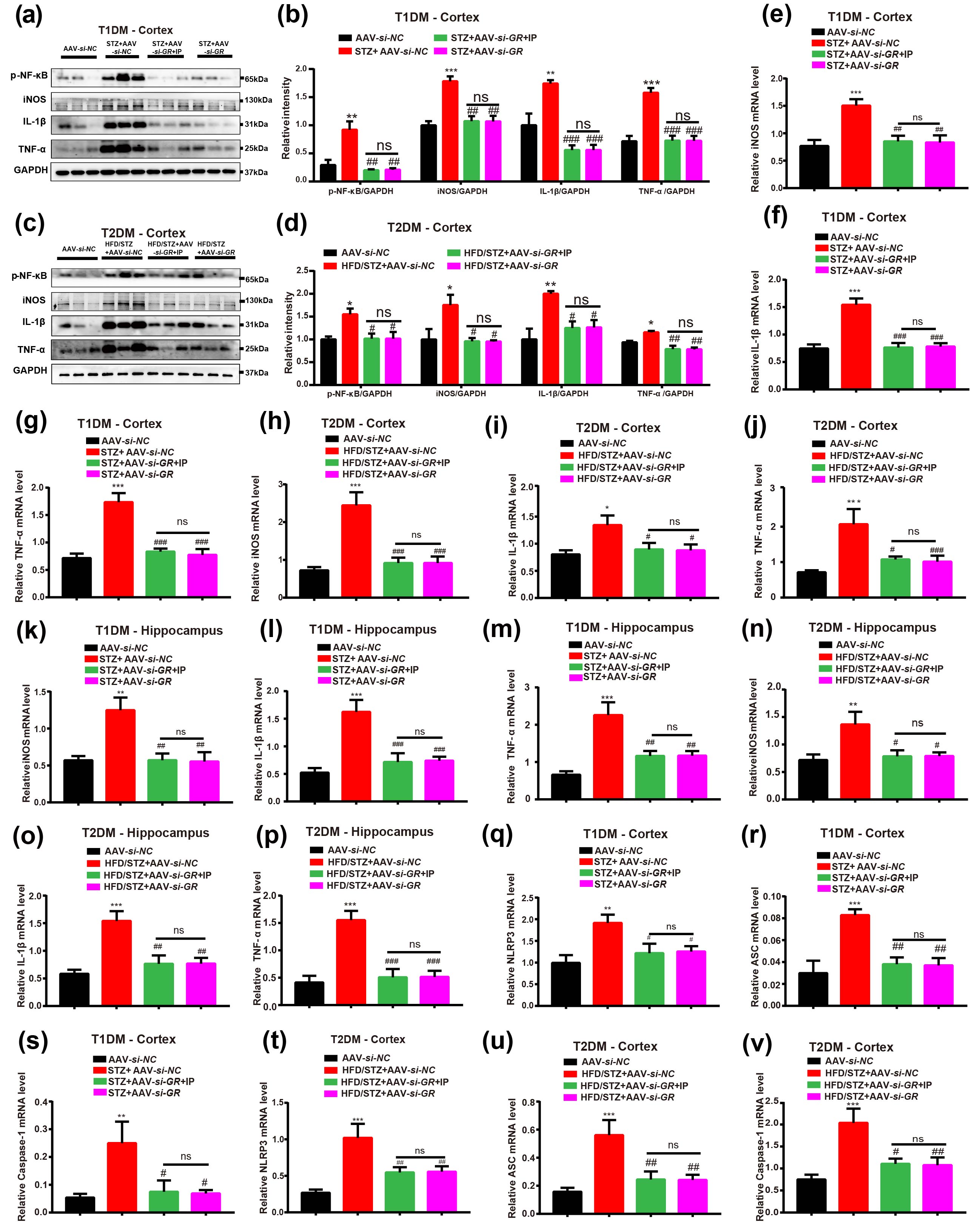

Supplement: Supplementary file 13 — Fig S13 [file ACEL-21-e13572-s009.jpg]
