# Supplementary material for: Ipriflavone as a non‐steroidal glucocorticoid receptor antagonist ameliorates diabetic cognitive impairment in mice
Source: Aging Cell. 2022 Feb 16;21(3):e13572. doi: 10.1111/acel.13572 (PMC8920458; doi:10.1111/acel.13572)
Supplement: Supplementary file 14 — Supplementary Material [file ACEL-21-e13572-s012.docx]

**Materials and Methods**

**Materials**

Ipriflavone (IP) was purchased from Target Molecule Corp (USA). Dexamethasone (DEX), mifepristone (Mife), dimethy1 sulfoxide (DMSO), Tween-80, 3-(4,5-dimethylthiazol-2-yl)-2,5-diphenyltetrazolium bromide (MTT), DNase, poly-D-lysine (PDL), streptozotocin (STZ), lipopolysaccharide (LPS), palmitic acid (PA) and pyrrolidinedithiocarbamate ammonium (PDTC) were from Sigma-Aldrich (St, Louis, Missouri USA). *si-NC* and *si-GR* were from GenePharma Co,.Ltd. AAV-ePHP-*si*-GR was established by GENE Co,.Ltd. Antibodies against PI3K, p-PI3K, AKT, p-AKT, NF-κB, p-NF-κB, L-1β, iNOS, TNF-α, PSD95, Synaptophysin (SYN), VAMP2, tau, p^9^-GSK3β, GSK3β, TrkB, CREB and p-CREB were from Cell Signaling Technology (Danvers, MA, USA). Antibodies against p-TrkB, p^396^-tau, p^199^-tau, NLRP3 and BDNF were from Abcam (Cambridge, UK). All cell culture reagents were purchased from Gibco (USA).

Plasmids of pCI-nGFP-C656G, pRL-SV40 and pUAS-TK-Luc were kindly donated respectively by Dr. Gordon Hager (National Cancer Institute, National Institutes of Health), Dr. J Larry Jameson (Department of Medicine, Northwestern Memorial Hospital) and Dr. Daniel P. Kelly (School of Medicine, Washington University). Other plasmids were available in our own lab.

**Cell culture**

U2OS/GR-GFP cells (Bioimage, Denmark) were cultured in DMEM/F12 medium containing 10% fetal bovine serum (FBS) and 100 U/ml penicillin-streptomycin (PS).

HEK293T cells were cultured with DMEM/High glucose culture medium (supplemented with 10% FBS and 100U/ml PS).

Primary neurons were isolated from embryos of C57BL/6 mice (embryonic day 18). The brain was harvested immediately and minced into small pieces, then digested with D-Hank’s buffer containing 0.125% trypsin and 200 U/mL DNase for 15 min (37 °C) and stopped with DMEM/High glucose culture medium containing 10% FBS and 100 U/ml PS. Cells were seeded in 6 - well plates at 37 °C for 6 h and replaced into neurobasal medium supplemented with 2% B27, 0.5 μmol/L-glutamine and 50 U/mL PS for 9 d.

Primary microglia were obtained from newborn C57BL/6 mice within 24 h. The brain tissues were minced and digested in D-Hank’s buffer as described above. Cells were centrifuged (300 g, 10 min) and cultured in DMEM/F12 with a poly-D-lysine-coated 75cm^2^ flask (at a density of 2×10^5^cells/mL) for 7 days. Primary microglia were separated by shaking several times and harvested by centrifugation.

***siRNA* interference assay**

Cells were seeded in 12 well plates overnight and transfected with 50 pmol of *si-GR* or *si*-*NC* for 6 h. Then, the medium was replaced with DMEM and incubated for another 32 h. Finally, the cells were exposed to exutory and/or IP for 24 h ([Lu et al., 2021](#_ENREF_11)) (Figure S1a).

**Mammalian one-hybrid and transactivation assays**

HEK293T cell transfection assay was performed using Calcium Phosphate Cell Transfection kit (Beyotime, Haimen, Jiangsu, China) according to the manufacturer’s instruction ([Xu et al., 2018](#_ENREF_16)). Plasmids of pCMX-Gal4-GR-LBD, pUAS-TK-luc, pCI-nGFP-C656G (GR-GFP), pGL3-GRE-Luc and pRL-SV40 were transfected into cells. After transfection for 6 h, the cells were exposed to dexamethasone (DEX, known GR agonist) or mifepristone (mife, known GR antagonist) with or without IP for 24 h. Finally, cells were washed, and luciferase activity was detected by Dual-luciferase reporter assay system kit (Promega, Madison, Wisconsin, USA) (Figure S1b).

**MTT assay**

MTT assay was used to evaluate cell viability. The cells were exposed to exutory and/or IP for 24 h as previously described (Figure S1c) ([X. Li et al., 2020](#_ENREF_9); [Xu et al., 2020](#_ENREF_17)).

**Animal model**

C57BL/6 male mice were purchased from Beijing Charles River Experimental Animal Co., Ltd. (Beijing, China) and kept in the specific pathogen-free (SPF) facility in a regular 12:12 h light/dark cycle. All animal experiments were performed in compliance with the institutional ethical guidelines on animal care of Nanjing University of Chinese Medicine (No. 012071001560).

*T1DM mice-* T1DM mice were obtained based on the published approach ([Clodfelder-Miller et al., 2006](#_ENREF_2)). Briefly, 8-week-old C57BL/6 male mice were fed with normal diet for one week, and then randomly divided into negative control group (Control, n = 15) and diabetic model group (n = 75) given by an intraperitoneal injection (*i.p.*) with a single dose of STZ (150 mg/kg, Sigma-Aldrich) dissolved in citrate buffer. One week after injection, blood glucose levels of the mice were detected, and the mice with blood glucose level ≥16.5 mmol/L were defined as T1DM model with typical clinical hallmarks including increased thirst, extreme hunger, unintended weight loss and irritability ([Lee et al., 2018](#_ENREF_7)). Subsequently, T1DM mice were divided into vehicle group (STZ, n = 25), IP (20, 40 mg/kg) group (STZ+IP-20, 40; n = 25 per group) according to blood glucose level and body weight.

*T2DM mice*- T2DM mice were prepared according to the published approaches([X. Y. Li et al., 2018](#_ENREF_10); [Xu et al., 2018](#_ENREF_16)). After adapting for one week, 5-week-old C57BL/6 male mice were assigned to normal diet group (Control, n = 10) and high-fat diet (HFD) group (n = 45), which were fed with normal chow and high-fat diet (HFD, 58% fat, 17% carbohydrate and 25% protein; Research Diets, New Brunswick, NJ, USA) for 4 weeks to induce insulin resistance. Then, mice in HFD and control groups received an injection of STZ (100 mg/kg, *i.p.*) and the same volume of vehicle. One week after injection, the mice with blood glucose level ≥11.0 mmol/L were identified as a non-obesity-related subtype of T2DM ([Sauve et al., 2016](#_ENREF_14)). T2DM mice were then divided into vehicle group (HFD/STZ, n = 15), IP (20, 40 mg/kg) group (HFD/STZ+IP-20, 40; n = 15 per group). Mice were maintained with HFD until sacrificed.

*GR knockdown mice-* Brain-specific GR knockdown mice were prepared according to the published approaches ([Chan et al., 2017](#_ENREF_1); [Lu et al., 2021](#_ENREF_11)). Briefly, adeno-associated virus AAV-ePHP-*si-GR* or AAV-ePHP*-si-NC* was injected into experimental mice through tail vein (1×10^11^ genome copies/mouse). Two groups of wild type mice injected with AAV-ePHP*-si-NC* served as negative control groups (AAV-*si-NC*, n = 15 per group) in diabetic mice experiments. T1DM mice with AAV-ePHP*-si-NC* or AAV-ePHP-*si-GR* were divided into STZ+AAV-*si-NC*, STZ+AAV-*si-GR*+IP (40 mg/kg) and STZ+AAV-*si-GR* (n = 25 per group). T2DM mice with AAV-ePHP*-si-NC* or AAV-ePHP-*si-GR* were assigned to HFD/STZ+AAV-*si-NC*, HFD/STZ+AAV-*si-GR* +IP (40 mg/kg) and HFD/STZ+AAV-*si-GR* (n = 15 per group).

Mice were treated by gavage daily with IP or the same volume of vehicle (saline containing 2% DMSO and 5% Tween-80) for 8 weeks.

**Behavior test**

*Morris water maze-* Morris water maze (MWM) test was performed according to the published approach ([X. Li et al., 2020](#_ENREF_9); [Zhu et al., 2010](#_ENREF_19)). In brief, an apparatus consisted of four equal-sized quadrants with an escape platform submerged 1 cm in the center of the target quadrant was employed for training. Mice were allowed to seek the invisible platform within 60 s, otherwise guided to the platform by the handler, and stayed there for 10 s to memorize the environment. Mice were individually trained each day for continuous 5 days before the probe test conducted on day 6. For probe test, the hidden platform was removed, and mice were put into the quadrant. The escape latency in the target quadrant and crossing times of the tested mice were recorded in 60 s to assess their spatial memory capacity.

*Y-maze test*- The exploratory activity and spontaneous alternation of mice were evaluated by Y-maze test with an apparatus consisting of three arms (A, B and C) as described in previous reports ([Huang et al., 2015](#_ENREF_5); [Lei et al., 2021](#_ENREF_8)). At the first stage, one arm was blocked, and mice were allowed to explore the other two arms for 5 min. After 2 h, mice were allowed to move freely throughout all 3 arms for 5 min in the test stage. A video camera was used to record the movement of each mouse. The spontaneous alternation was calculated as spontaneous alternation score (%) = alternation number (entering three different arms, such as ABC/BCA/CAB) / (total number - 2) × 100%.

*Open-field test-* Open-field test (OFT) was used to evaluate the ability of exploration and anxiety-related behaviors in mice([Kuniishi et al., 2017](#_ENREF_6); [Pang et al., 2021](#_ENREF_13)). OFT was performed in 4 boxes (50 × 50 × 50 in cm) placed in a dim-light room. After 1 h acclimatization to the behavioral testing room, mice were placed in the center of the experimental box and allowed to explore freely for 5 min. Total movements in the open field such as distance travel and time spent in central area were recorded.

*New object recognition test*- New object recognition (NOR) test was commonly used to assess novel object exploration activity of mice ([Miedel et al., 2017](#_ENREF_12)). In the habituation trial, two objects with the same size, shape, material, and color were placed on the box bottom. Mice were placed at the center of box and allowed to explore freely for 15 min. After 4 h, one of the previous objects was replaced by a novel object and mice were returned to the box to explore freely for 5 min. The discrimination index (DI) data were analyzed. DI = (Times exploring novel object-Times exploring familiar object)/Total object exploration time.

**Hormonal measurement**

To examine the levels of HPA-axis hormones in mice serum, the concentrations of corticotropin-releasing hormone (CRH) and corticosterone (CORT) were quantified by using commercial enzyme-linked immunosorbent (ELISA) kits (Elabscience Biotechnology Co., Ltd. China). Each procedure was carried out according to the kit instructions.

**Western blot analysis**

Western blot assay was conducted as described in the published approaches ([Guo et al., 2017](#_ENREF_3); [X. Li et al., 2020](#_ENREF_9)). In brief, brain tissues or cells were lysed with RIPA buffer containing protease and phosphatase inhibitor cocktail and protein samples were quantified with BCA protein quantitative kit (Beyotime). Samples were separated by SDS-PAGE and transferred into a nitrocellulose membrane. After blocking, membranes were probed with corresponding antibodies. The protein bands were visualized by ChemiDoc^TM^MP Imaging System (BIO-RAD CO, USA) and quantified using Image J, normalized to GAPDH (Thermo).

**Quantitative real-time PCR analysis**

Total RNA from mouse brains and cells were extracted with TRIzol (TAKARA Bio, Japan) according to the manufacturer’s protocol([Xu et al., 2015](#_ENREF_18)). 1 µg of RNA was used to synthesize cDNA by the commercial Prime Script RT reagent kit (TaKaRa Biotechnology Co). Quantitative real-time PCR (qPCR) was performed by using SYBR Premix Ex Taq kit (TaKaRa Bio, Japan) and BIO-RAD CFX connect real-time system (BIO-RAD CO, USA). Relative expressions of targeted genes were analyzed by using the 2^-ΔΔCt^ method normalized to GAPDH.

The primers were synthesized by Sangon Biotech (Shanghai, China) as follows:

iNOS (F), CTTGGTAGGTGGCTATGTGGCATC;

| iNOS (R), GGCTGTGAGGTGAGGTTGAAGAAG; |
| --- |

TNF-α (F), CCCACGTCGTAGCAAACCACCA;

TNF-α (R), CCATTGGCCAGGAGGGCGTTG;

IL-1β (F), GCAGCAGCACATCAACAAGAGC;

IL-1β (R), TGTCCTCATCCTGGAAGGTCCACG;

NLRP3 (F), AGAGAGGAGAGGAGGTCGAC

NLRP3 (R), AGAGAGGAGAGGAGGTCGAC

ASC (F), CCCATAGACCTCACTGATAAAC

ASC (R), AGAGCATCCAGCAAACCA

Caspase-1 (F), AACCACTCGTACACGTCTTGC

Caspase-1 (R), ATCCTCCAGCAGCAACTTCA

GAPDH (F), ACAGCAACAGGGTGGTGGAC;

GAPDH (R), TTTGAGGGTGCAGCGAACTT.

**Immunofluorescence assay**

Immunofluorescence assay was conducted as described in the published approach ([X. Li et al., 2020](#_ENREF_9)). For mouse brains, samples were harvested and fixed in paraformaldehyde at room temperature for 24 h followed by dehydration in sucrose for 48 h. Brain tissues were embedded with optimal cutting temperature (OCT) and sectioned (20 µm) by cryomicrotome (Leica, Germany). For cells, samples were fixed with paraformaldehyde at room temperature for 25 min and washed gently 3 times. After permeating by Triton X-100 (10 min), samples were blocked with 5% BSA (30 min) and incubated with primary antibody (1:500) overnight at 4 ℃. Tissue sections or cells were then incubated with second antibody (1:800) for 1 h at room temperature. After being rinsed, samples were incubated with DAPI (1:200) for 10 min at room temperature. Fluorescence images were captured by the confocal laser scanning microscopy (Leica, Germany) and analyzed by the Image J software.

**AT8 staining**

Since AT8 staining assay is believed to be able to recognize phosphorylated paired-helical filament tau (PHF-tau) at sites of serine 202 and threonine 205 without cross-reactivity with unphoshorylated tau ([Holmes et al., 2014](#_ENREF_4)), AT8 staining assay was performed.

In the assay, brain slides (20 μm) were incubated with 5% triton X-100 for 10 min and blocked in 5% BSA for 30 min at room temperature, followed by incubation with AT8 antibody (1:250, Invitrogen, St, Louis, Missouri, USA) overnight at 4 °C. After washed 3 times, slides were incubated with the secondary antibody for 1 h and stained with diaminobenzidine (DAB) for 5 min. Images were obtained by an automated upright microscope.

**Golgi staining**

Morphology of dendritic spines in the brain was assayed by a commercial Kit (FD Neuro technologies, Elliot City, MD, USA) according to the manufacture’s protocols ([Wang et al., 2016](#_ENREF_15)). In brief, the collected tissues were immersed in solution A and B for 14 days, and then replaced with solution C for further 7 days. Subsequently, the brains were embedded in OCT and sectioned (100 µm, coronal). Frozen brain sections were stained with solution D and E, and dehydrated by 50%, 75%, 95% and 100% ethanol.

**Electrophysiology assay**

Mice were deeply anesthetized with sodium pentobarbital, and brain was then removed rapidly into ice-cold chilled artificial cerebrospinal fluid (ACSF). Brains were sectioned transversely (400 μm) and incubated into a chamber filled with ACSF in an oxygenated environment (95% O_2_, 5% CO_2_) for 30 min. After incubation, brain slices were transferred into a chamber and immersed in the oxygenated ACSF. A baseline of fEPSP was recorded for 10 min, and long‐term potentiation (LTP) was induced by high frequency stimulation (HFS, 1s, 100 HZ) and recorded for 60 min. Data were recorded and analyzed by pCLAMP 9.0 (Axon Instruments, USA).

**References:**

Chan, K. Y., Jang, M. J., Yoo, B. B., Greenbaum, A., Ravi, N., Wu, W. L., et al. (2017). Engineered AAVs for efficient noninvasive gene delivery to the central and peripheral nervous systems. *Nat Neurosci,* ***20***, 1172-1179. doi:10.1038/nn.4593

Clodfelder-Miller, B. J., Zmijewska, A. A., Johnson, G. V., & Jope, R. S. (2006). Tau is hyperphosphorylated at multiple sites in mouse brain in vivo after streptozotocin-induced insulin deficiency. *Diabetes, 55*(12), 3320-3325. doi:10.2337/db06-0485

Guo, X. D., Sun, G. L., Zhou, T. T., Wang, Y. Y., Xu, X., Shi, X. F., et al. (2017). LX2343 alleviates cognitive impairments in AD model rats by inhibiting oxidative stress-induced neuronal apoptosis and tauopathy. *Acta Pharmacol Sin, 38*(8), 1104-1119. doi:10.1038/aps.2016.128

Holmes, B. B., Furman, J. L., Mahan, T. E., Yamasaki, T. R., Mirbaha, H., Eades, W. C., et al. (2014). Proteopathic tau seeding predicts tauopathy in vivo. *Proc Natl Acad Sci U S A, 111*(41), E4376-E4385. doi:10.1073/pnas.1411649111

Huang, H., Wang, L., Cao, M., Marshall, C., Gao, J., Xiao, N., et al. (2015). Isolation Housing Exacerbates Alzheimer's Disease-Like Pathophysiology in Aged APP/PS1 Mice. *Int J Neuropsychopharmacol, 18*(7), pyu116. doi:10.1093/ijnp/pyu116

Kuniishi, H., Ichisaka, S., Yamamoto, M., Ikubo, N., Matsuda, S., Futora, E., et al. (2017). Early deprivation increases high-leaning behavior, a novel anxiety-like behavior, in the open field test in rats. *Neurosci Res, 123*, 27-35. doi:10.1016/j.neures.2017.04.012

Lee, Y. S., Lee, C., Choung, J. S., Jung, H. S., & Jun, H. S. (2018). Glucagon-Like Peptide 1 Increases beta-Cell Regeneration by Promoting alpha- to beta-Cell Transdifferentiation. *Diabetes, 67*(12), 2601-2614. doi:10.2337/db18-0155

Lei, T., Yang, Z., Xia, X., Chen, Y., Yang, X., Xie, R., et al. (2021). A nanocleaner specifically penetrates the blood‒brain barrier at lesions to clean toxic proteins and regulate inflammation in Alzheimer’s disease. *Acta Pharmaceutica Sinica B*. doi:10.1016/j.apsb.2021.04.022

Li, X., Lu, J., Xu, Y., Wang, J., Qiu, X., Fan, L., et al. (2020). Discovery of nitazoxanide-based derivatives as autophagy activators for the treatment of Alzheimer's disease. *Acta Pharm Sin B, 10*(4), 646-666. doi:10.1016/j.apsb.2019.07.006

Li, X. Y., Lu, S. S., Wang, H. L., Li, G., He, Y. F., Liu, X. Y., et al. (2018). Effects of the fenugreek extracts on high-fat diet-fed and streptozotocin-induced type 2 diabetic mice. *Animal Model Exp Med, 1*(1), 68-73. doi:10.1002/ame2.12004

Lu, J., Zhang, C., Lv, J., Zhu, X., Jiang, X., Lu, W., et al. (2021). Antiallergic drug desloratadine as a selective antagonist of 5HT(2A) receptor ameliorates pathology of Alzheimer's disease model mice by improving microglial dysfunction. *Aging Cell, 20*(1), e13286. doi:10.1111/acel.13286

Miedel, C. J., Patton, J. M., Miedel, A. N., Miedel, E. S., & Levenson, J. M. (2017). Assessment of Spontaneous Alternation, Novel Object Recognition and Limb Clasping in Transgenic Mouse Models of Amyloid-β and Tau Neuropathology. *J Vis Exp*(123). doi:10.3791/55523

Pang, L., Zhu, S., Ma, J., Zhu, L., Liu, Y., Ou, G., et al. (2021). Intranasal temperature-sensitive hydrogels of cannabidiol inclusion complex for the treatment of post-traumatic stress disorder. *Acta Pharmaceutica Sinica B*. doi:10.1016/j.apsb.2021.01.014

Sauve, M., Hui, S. K., Dinh, D. D., Foltz, W. D., Momen, A., Nedospasov, S. A., et al. (2016). Tumor Necrosis Factor/Sphingosine-1-Phosphate Signaling Augments Resistance Artery Myogenic Tone in Diabetes. *Diabetes, 65*(7), 1916-1928. doi:10.2337/db15-1450

Wang, S., Yu, L., Yang, H., Li, C., Hui, Z., Xu, Y., et al. (2016). Oridonin attenuates Synaptic Loss and Cognitive Deficits in an Abeta1-42-Induced Mouse Model of Alzheimer's Disease. *PLoS One, 11*(3), e0151397. doi:10.1371/journal.pone.0151397

Xu, X., Shi, X., Chen, Y., Zhou, T., Wang, J., Xu, X., et al. (2018). HS218 as an FXR antagonist suppresses gluconeogenesis by inhibiting FXR binding to PGC-1α promoter. *Metabolism, 85*, 126-138. doi:10.1016/j.metabol.2018.03.016

Xu, X., Xu, X., Hao, Y., Zhu, X., Lu, J., Ouyang, X., et al. (2020). Antispasmodic drug drofenine as an Inhibitor of Kv2.1 channel ameliorates peripheral neuropathy in diabetic mice. *iScience, 23*(10), 101617. doi:10.1016/j.isci.2020.101617

Xu, X., Xu, X., Liu, P., Zhu, Z. Y., Chen, J., Fu, H. A., et al. (2015). Structural basis for small molecule NDB (N-Benzyl-N-(3-(tert-butyl)-4-hydroxyphenyl)-2,6-dichloro-4-(dimethylamino) Benzamide) as a selective antagonist of farnesoid X receptor alpha (FXRalpha) in stabilizing the homodimerization of the receptor. *J Biol Chem, 290*(32), 19888-19899. doi:10.1074/jbc.M114.630475

Zhu, Z. Y., Li, C. J., Wang, X., Yang, Z. Y., Chen, J., Hu, L. H., et al. (2010). 2,2',4'-Trihydroxychalcone from Glycyrrhiza glabra as a new specific BACE1 inhibitor efficiently ameliorates memory impairment in mice. *Journal of Neurochemistry, 114*(2), 374-385. doi:10.1111/j.1471-4159.2010.06751.x
